# Reversible Optical Switching of Polyoxovanadates and Their Communication via Photoexcited States

**DOI:** 10.1002/advs.202401595

**Published:** 2024-06-13

**Authors:** Eric Vogelsberg, Jan Griebel, Iryna Engelmann, Jens Bauer, Florian Taube, Björn Corzilius, Stefan Zahn, Axel Kahnt, Kirill Yu. Monakhov

**Affiliations:** ^1^ Leibniz Institute of Surface Engineering (IOM) Permoserstr. 15 04318 Leipzig Germany; ^2^ Institute of Chemistry and Department of Life Light & Matter University of Rostock Albert‐Einstein‐Str. 25–27 18059 Rostock Germany; ^3^ Leibniz‐Institute of Catalysis (LIKAT) Albert‐Einstein‐Str. 29a 18059 Rostock Germany

**Keywords:** charge transfer, electronic structure, excited states, irradiation, polyoxometalates

## Abstract

The 2‐bit Lindqvist‐type polyoxometalate (POM) [V_6_O_13_((OCH_2_)_3_CCH_2_N_3_)_2_]^2–^ with a diamagnetic {V_6_O_19_} core and azide termini shows six fully oxidized V^V^ centers in solution as well as the solid state, according to ^51^V NMR spectroscopy. Under UV irradiation, it exhibits reversible switching between its ground S_0_ state and the energetically higher lying states in acetonitrile and water solutions. TD‐DFT calculations demonstrate that this process is mainly initialized by excitation from the S_0_ to S_9_ state. Pulse radiolysis transient absorption spectroscopy experiments with a solvated electron point out photochemically induced charge disproportionation of V^V^ into V^IV^ and electron communication between the POM molecules via their excited states. The existence of this unique POM‐to‐POM electron communication is also indicated by X‐ray photoelectron spectroscopy (XPS) studies on gold‐metalized silicon wafers (Au//SiO_2_//Si) under ambient conditions. The amount of reduced vanadium centers in the “confined” environment increases substantially after beam irradiation with soft X‐rays compared to non‐irradiated samples. The excited state of one POM anion seems to give rise to subsequent electron transfer from another POM anion. However, this reaction is prohibited as soon as the relaxed T_1_ state of the POM is reached.

## Graphical abstract

**POM‐to‐POM electron communication**. Pulse radiolysis, quantum chemistry calculations, solution photochemistry, and irradiation studies of solid samples by soft X‐rays demonstrate that Lindqvist‐type hexavanadate structures can switch and interact with each other under certain irradiation conditions using their excited states, thus opening new possibilities for polyoxometalates in various fields of science.

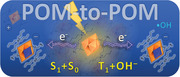

## 1 Introduction

Electron‐exchange reactions between reduced and oxidized polyoxometalate (POM) anions and the electron transfer between POMs and organometallic complexes or metal–organic frameworks have attracted much attention due to their exceptional role in various catalytic processes.^[^ ^1^, ^2^, ^3^, ^4^, ^5^, ^6^ ^]^ Recently, photoinduced electron transfer from the excited state of the [Mo_6_I_8_Cl_6_]^2−^ cluster to POMs was also observed.^[^ ^7^ ^]^ In addition to the far‐reaching potential of POMs in oxidative as well as electro‐ and photocatalytic systems, observation and controlled programming of intermolecular electron interactions triggered by the photochemistry/excited state physics of POMs may provide access to the development of POM‐based biomimetic functionalities, out‐of‐equilibrium supramolecular assembles, information processing, and artificial neural networks that can be driven by natural fuels such as light and electricity.^[^ ^8^ ^]^

POMs are soluble, redox‐active metal–oxo cluster anions, which are charge‐balanced by cations.^[^ ^9^ ^]^ Insights into the chemistry of POMs in solution or on electrode surfaces shows that POM countercations take part in or even modulate electron transfer processes within and across POM‐based ground‐state systems.^[^ ^10^, ^11^, ^12^, ^13^, ^14^ ^]^ Their electrical conductance can be switched, for example, by the electric field of a scanning tunneling microscope (STM). In the absence of any external stimulus, reducing this “POM–cations–POM” interaction to direct electron interaction between POM anions is, however, elusive, since countercations are always present in solution or on surfaces when POMs are deposited by using low‐cost drop casting or dip/spin coating methods. The elegant ways to induce direct communication between POMs and, thus, allow them to exchange information stored in their charge and spin states via multielectron transfers,^[^ ^15^ ^]^ like cofactors in enzymatic systems, would be threefold: i) by cross‐linking POM units through their conjugation with ground‐state (through space) charge‐transfer polymers;^[^ ^16^, ^17^ ^]^ ii) by using the mass‐selective ion soft‐landing technique to remove bulky cations from deposition solutions and to bind ground‐state molecular ions of same polarity,^[^ ^18^, ^19^ ^]^ generated in the gas phase, on surfaces (*cf*. the anion–anion chemistry of borates)^[^ ^20^ ^]^; iii) by intermolecular reactions between neighboring photoexcited states of POMs (*cf*. the behavior of fullerenes in excited states).^[^ ^21^ ^]^ Indeed, these design parameters for ground‐state POM structures may come with many challenges such as, for example, inhibited intermolecular interactions of POMs due to covalently attached ligand spacers or Coulomb repulsion forces between anionic POM single units or anionic POM building blocks of self‐assembled materials.^[^ ^22^, ^23^ ^]^ However, parameter iii (also in combination with i or ii) offers a promising platform for inducing, programming, and spatially controlling the electrical conductance and the fast light‐driven multistate switching of POMs via their stimuli‐responsive redox states.^[^ ^24^ ^]^ To our knowledge no data have been reported so far for such excited state physics of POM‐to‐POM interactions in unconfined and confined environments.

Among synthetic POM capacitors, especially polyoxovanadates with the biologically significant V^V^/V^IV^ redox couple VO_2_^+^/VO^2+^ were identified as highly photo‐sensitive anions,^[^ ^25^, ^26^, ^27^, ^28^ ^]^ recognized as quantum cellular automata,^[^ ^29^, ^30^ ^]^ and established as potential‐induced multistate switches at room temperature (r.t.) at the levels of single molecules and thin films.^[^ ^31^, ^32^ ^]^ Combining and exploiting these underlying properties in a single polyoxovanadate compound is a challenging task. However, this might create new advanced smart materials with useful functions at the cross‐section between chemistry, biology, and engineering. For example, one of the exciting questions to be investigated is whether these POMs, with their charge/spin‐active vanadium centers susceptible to applied electrical and light stimuli, can in principle tunnel electrons across an artificial polyoxovanadate redox chain, replacing common cofactors such as iron porphyrins and iron–sulfur clusters that carry out multielectron transfers in enzymes at the mitochondrial membrane.^[^ ^33^ ^]^ Another question is whether these POMs can serve as active components of optoelectronic memristive cells for energy‐efficient in‐memory and brain‐inspired neuromorphic computing with ultra‐fast data transfer.^[^ ^34^ ^]^

In this Article, we demonstrate a reversible, photoinduced switching and electron processing between tris(alkoxo)‐supported Lindqvist‐type hexavanadates (POV6) via their V^V^ (3d^0^) and V^IV^ (3d^1^) centers. The intermolecular interaction of these anions arises by the excitation from the ground state of the POV6 to its excited states that initialize in turn subsequent reactions with neighboring POV6 anions under beam irradiation using UV light or soft X‐rays. The obtained data were evaluated from nuclear magnetic spectroscopy (NMR), ultraviolet‐visible (UV/vis) spectroscopy, pulse radiolysis transient absorption spectroscopy, X‐ray photoelectron spectroscopy (XPS), and quantum chemistry calculations.

## 2 Results and Discussion

### 2.1 Irradiation of Photoactive POM in Solution

**Figure 1:**
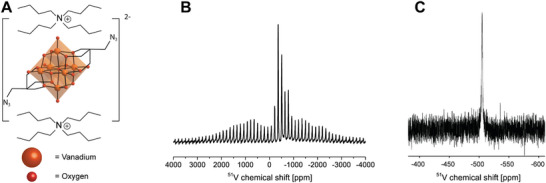
A) Structure of the [V_6_O_13_((OCH_2_)_3_CCH_2_N_3_)_2_]^2–^ anion charge‐balanced by two nBu_4_N^+^ cations. B) ^51^V MAS‐NMR spectrum in the solid state at r.t. C) ^51^V NMR spectrum in solution at r.t.

**Figure 2:**
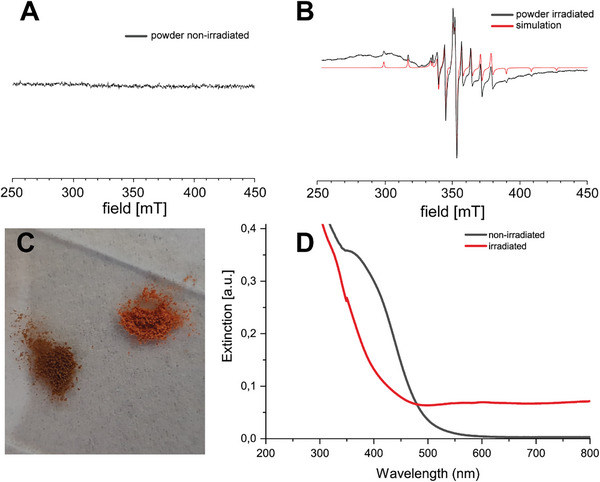
A) EPR in the solid state before irradiation. B) EPR after irradiation. C) Comparison of the powder samples (left: after irradiation, right: before). D) UV/vis in solution before and after irradiation.

The title compound (*n*Bu_4_N)_2_[V_6_O_13_((OCH_2_)_3_CCH_2_N_3_)_2_] (POV6, **Figure** **1**A) was synthesized according to a synthetic protocol published in the literature.^[^ ^35^ ^]^ The ^51^V magic angle spinning (MAS) NMR spectrum of the solid‐state sample (Figure 1B) and the ^51^V NMR spectrum of the sample (Figure 1C) dissolved in CD_3_CN show, respectively, singlet peaks at *δ* = −511 ppm and *δ*  = −505 ppm indicating the presence of only fully‐oxidized vanadium(V) centers. This degree of oxidation of the compound is further confirmed by the EPR‐silent non‐irradiated powder sample (**Figure** **2**C).

A 90 s irradiation period of the compound with light at 365 nm (16 W LED, 30% power at a distance of 30 mm) causes it to change color from orange to brown in the powder (Figure 2B). In solution (Figure 2F), the color changes from yellow to green or, with longer irradiation periods, even to blue. This change is also detected in the absorption spectrum (Figure 2D), where for the irradiated sample an increase in the baseline absorbance in the range above 450 nm is observed, as well as a decrease in the local maximum at 400 nm existing in the non‐irradiated sample. The observation can be explained by the presence of V^4+^ after irradiation, as shown in the EPR spectrum in Figure 2A. Although the redox state of vanadium atoms in the compound changes after irradiation, no changes in the residual structure of the POV6 is detected in ^51^V and ^1^H NMR spectra (Figures S1 and S2, Supporting Information). Irradiation of identical samples with different wavelengths ranging from 350 to 475 nm shows that the change in the absorption spectrum, and therefore, probably also the amount of V^4+^ produced, depends on the wavelength, and shows the strongest effect at 375 nm (Figure S4, Supporting Information). Observation of the dissolved POV6 sample over the next 3.5 h after irradiation also shows that the observed change appears to be reversible under existing conditions, as the UV/vis spectra slowly return to the original pre‐irradiation state (Figure S5, Supporting Information). Since the initial change correlates with a reduction of vanadium from +V to +IV, it seems reasonable to assume that this process is associated with the oxidation of vanadium centers back to their pristine +V state.

Differences in the UV/vis spectra of two samples (*n*Bu_4_N)_2_[V_6_O_13_((OCH_2_)_3_CCH_2_N_3_)_2_] irradiated in 100%‐vol MeCN and 20%‐vol MeCN solution indicate that in addition to wavelength, also the chemical surrounding influences the formation of V^IV^ inside the POV6 and the subsequent oxidation process. The recorded data clearly demonstrate that the UV/vis spectrum of the sample in an aqueous medium undergoes a change back to the non‐irradiated state in a shorter period of time than its counterpart in a purely organic medium (Figure S5, Supporting Information).

### 2.2 Computational Studies

**Figure 3:**
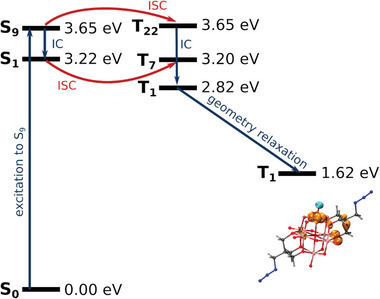
Energy levels of excited states obtained by TD‐DFT in the S_0_ ground state geometry except for the optimized T_1_ ground state to the right. The [V_6_O_13_((OCH_2_)_3_CCH_2_N_3_)_2_]^2–^ anion shows the T_1_ ground state geometry with isosurface of increased *α* (orange) and *β* (cyan) spin density.

The properties of the exited states of POV6 {V_6_^V^} were investigated by time‐dependent density functional theory (TD‐DFT) calculations (**Figure** **3**). The transition dipole moment between S_0_ and S_1_ is close to zero (0.00 a.u.), while it is 0.52 a.u. for the S_0_ to S_9_ excitation. Thus, an initial excitation into the S_9_ state seems most likely. Subsequently, internal conversion will relax the system into the S_1_ state. However, a fast intersystem crossing (ISC) to a triplet state cannot be excluded since the unique nature of the POV6 {V_6_^V^} results in many triplet states with nearly vanishing energy gap between the excited singlet and triplet states. This should support fast intersystem crossing (ISC) rates from a singlet to the triplet state, which can relax into the T_1_ ground state structure.

#### 2.2.1 Redox Reactions in Acetonitrile Solution

 

(1) $$\begin{array}{ccc} & & \mathrm{POV}6\left\{{V_{6}}^{V}\right\}\left(S1\right)+\mathrm{MeCN}\to \mathrm{POV}6\left\{{V_{5}}^{V}V^{\mathrm{IV}}\right\}\\ & & \;+\mathrm{MeC}N^{+}\;\;\Delta G:188.1\;\mathrm{kJ}\mathrm{mo}l^{-1} \end{array}$$

(2) $$\begin{array}{ccc} & & \mathrm{POV}6\left\{{V_{6}}^{V}\right\}\left(S1\right)+{N_{4444}}^{+}\to \mathrm{POV}6\left\{{V_{5}}^{V}V^{\mathrm{IV}}\right\}\\ & & \;+{N_{4444}}^{2+}\;\;\Delta G:76.8\;\mathrm{kJ}\mathrm{mo}l^{-1} \end{array}$$

(3) $$\begin{array}{ccc} & & \mathrm{POV}6\left\{{V_{6}}^{V}\right\}\left(S1\right)+\mathrm{POV}6\left\{{V_{6}}^{V}\right\}\left(S_{0}\right)\to \mathrm{POV}6\left\{{V_{5}}^{V}V^{\mathrm{IV}}\right\}\\ & & \;+\mathrm{POV}6{\left\{{V_{6}}^{V}\right\}}^{+}\;\;\Delta G:-98.9\;\mathrm{kJ}\mathrm{mo}l^{-1} \end{array}$$

(4) $$\begin{array}{ccc} & & \mathrm{POV}6\left\{{V_{6}}^{V}\right\}\left(T_{1}\right)+\mathrm{POV}6\left\{{V_{6}}^{V}\right\}\left(S_{0}\right)\to \mathrm{POV}6\left\{{V_{5}}^{V}V^{\mathrm{IV}}\right\}\\ & & \;+\mathrm{POV}6{\left\{{V_{6}}^{V}\right\}}^{+}\;\;\Delta G:55.5\;\mathrm{kJ}\mathrm{mo}l^{-1} \end{array}$$

The POV6 {V_6_^V^} compound is photochemically reduced in acetonitrile solution. Neither acetonitrile nor tetrabutylammonium (N_4444_^+^) can transfer an electron to the excited S_1_ state since these reactions are endergonic, Δ*G* = 188.1 kJ mol^−1^ and Δ*G* = 76.8 kJ mol^−1^, respectively. It seems only reasonable that a second POV6 {V_6_^V^} is oxidized by the S_1_ state of POV6 {V_6_^V^} (Δ*G* = −98.9 kJ mol^−1^). The energy level of the T_1_ state of POV6 {V_6_^V^} relaxed to its ground‐state geometry is already too low to oxidize a second POV6 {V_6_^V^} (Δ*G* = 55.5 kJ mol^−1^). Thus, the electron exchange between both complexes must occur very fast bevor the relaxed T_1_ ground state is reached.

#### 2.2.2 H‐Atom Transfer in Acetonitrile Solution

 

(5) $$\begin{array}{ccc} & & \mathrm{POV}6\left\{{V_{6}}^{V}\right\}\left(T_{1}\right)+\mathrm{MeCN}\to \mathrm{POV}6H\left\{{V_{5}}^{V}V^{\mathrm{IV}}\right\}\\ & & \;+•CH_{2}\mathrm{CN}\;\;\begin{array}{c} \Delta G:-34.0\;\mathrm{kJ}\mathrm{mo}l^{-1}\\ E_{A}:96.4\;\mathrm{kJ}\mathrm{mo}l^{-1} \end{array} \end{array}$$

(6) $$\begin{array}{ccc} & & \mathrm{POV}6\left\{{V_{6}}^{V}\right\}\left(T_{1}\right)+{N_{4444}}^{+}\to \mathrm{POV}6H\left\{{V_{5}}^{V}V^{\mathrm{IV}}\right\}\\ & & \;+•{N_{4444}}^{+}\;\;\begin{array}{c} \Delta G:-45.8\;\mathrm{kJ}\mathrm{mo}l^{-1}\\ E_{A}:108.2\;\mathrm{kJ}\mathrm{mo}l^{-1} \end{array} \end{array}$$

A second possibility might be an H‐atom transfer from acetonitrile to the T_1_ state of POV6 {V_6_^V^}. While this reaction is exergonic (ΔG = −34.0 kJ mol^−1^), the activation energy E_A_ for this process is 96.4 kJ mol^−1^, which is too high for a reaction at r.t. Also, an abstraction of the H‐atom from the tetrabutylammonium cation seems unlikely due to the high activation barrier (E_A_ = 108.2 kJ mol^−1^). Thus, solely a fast electron transfer from a S_0_ POV6 {V_6_^V^} ground‐state complex to an excited state of a second POV6 {V_6_^V^} complex before it reaches the T_1_ relaxed geometry seems reasonable in acetonitrile solution at r.t.

#### 2.2.3 Redox Reactions in Water

In water solution, a second reaction pathway was identified. While an electron transfer from water to the S_1_ state of POV6 {V_6_^V^} is unlikely (Δ*G* = 188.1 kJ mol^−1^), a reduction of a hydroxyl anion by the T_1_ state of POV6 {V_6_^V^} is exergonic (Δ*G* = −51.0 kJ mol^−1^). The reaction is still exergonic (Δ*G* = −11.1 kJ mol^−1^) if a •OH concentration of 1 mol L^−1^ at a pH of 7 is assumed. Thus, a reduction of a hydroxyl anion to a hydroxyl radical by the T_1_ state of POV6 {V_6_^V^} complex seems likely in water solution resulting in a reduced POV6 {V_5_^V^V^IV^} complex.

Finally, the computational studies indicate that the POV6 {V_5_^V^V^IV^} complex can be generated with a water solvated electron *e*_aq_^−^ (Δ*G* = −262.9 kJ mol^−1^) or (CH_3_)_2_•C(OH) (Δ*G* = −76.0 kJ mol^−1^), providing reaction pathways to generate POV6 {V_5_^V^V^IV^} with the S_0_ ground state of POV6 {V_6_^V^}.

(7) $$\begin{array}{ccc} & & \mathrm{POV}6\left\{{V_{6}}^{V}\right\}\left(S1\right)+\mathrm{POV}6\left\{{V_{6}}^{V}\right\}\left(S_{0}\right)\to \mathrm{POV}6\left\{{V_{5}}^{V}V^{\mathrm{IV}}\right\}\\ & & \;+\mathrm{POV}6{\left\{{V_{6}}^{V}\right\}}^{+}\;\;\;\;\;\Delta G:-140.0\;\mathrm{kJ}\mathrm{mo}l^{-1} \end{array}$$

(8) $$\begin{array}{ccc} & & \mathrm{POV}6\left\{{V_{6}}^{V}\right\}\left(T1\right)+\mathrm{POV}6\left\{{V_{6}}^{V}\right\}\left(S_{0}\right)\to \mathrm{POV}6\left\{{V_{5}}^{V}V^{\mathrm{IV}}\right\}\\ & & \;+\mathrm{POV}6{\left\{{V_{6}}^{V}\right\}}^{+}\;\;\;\;\Delta G:27.5\;\mathrm{kJ}\mathrm{mo}l^{-1} \end{array}$$

(9) $$\begin{array}{ccc} & & \mathrm{POV}6\left\{{V_{6}}^{V}\right\}\left(S1\right)+H_{2}O\to \mathrm{POV}6\left\{{V_{5}}^{V}V^{\mathrm{IV}}\right\}\\ & & \;+H_{2}O^{+}\;\;\;\;\;\;\;\;\;\Delta G:188.1\;\mathrm{kJ}\mathrm{mo}l^{-1} \end{array}$$

(10) $$\begin{array}{ccc} & & \mathrm{POV}6\left\{{V_{6}}^{V}\right\}\left(T_{1}\right)+OH^{-}\to \mathrm{POV}6\left\{{V_{5}}^{V}V^{\mathrm{IV}}\right\}\\ & & \;+•\mathrm{OH}\;\;\;\;\;\;\;\;\;\;\Delta G:-51.0\;\mathrm{kJ}\mathrm{mo}l^{-1} \end{array}$$

(11) $$\begin{array}{ccc} & & \mathrm{POV}6\left\{{V_{6}}^{V}\right\}\left(T_{1}\right)+{e_{\mathrm{aq}}}^{-}\to \mathrm{POV}6\left\{{V_{5}}^{V}V^{\mathrm{IV}}\right\}\\ & & \;\Delta G:-262.9\;\mathrm{kJ}\mathrm{mo}l^{-1} \end{array}$$

(12) $$\begin{array}{ccc} & & \mathrm{POV}6\left\{{V_{6}}^{V}\right\}+{\left(CH_{3}\right)}_{2}•\mathrm{COH}+H_{2}O\to \mathrm{POV}6\left\{{V_{5}}^{V}V^{\mathrm{IV}}\right\}\\ & & \;+{\left(CH_{3}\right)}_{2}\mathrm{CO}+H_{3}O^{+}\to \Delta G:-76.0\;\mathrm{kJ}\mathrm{mo}l^{-1} \end{array}$$

### 2.3 Radiation Chemical and Photochemical Studies

Pulse radiolysis studies under radiolytic reducing conditions and photochemical studies were conducted to experimentally identify the electron exchange pathway in dissolved hexavanadate compounds with a fully oxidized vanadium(V)–oxo cluster core, which can react as an electron acceptor. Additionally, these experiments were used to verify the reaction pathways suggested by the computational studies and to provide evidence for the potential reduction of OH^−^ to •OH.

For the pulse radiolysis study under reducing conditions, two different approaches were used. In the first approach solvated electrons (*e*_aq_^−^) were generated and probed as very powerful reductants (−2.7 V vs NHE).^[^ ^36^ ^]^ This study required N_2_ saturated dilute solutions of POV6 containing 5 vol% t‐butanol. Such conditions lead to the formation of the products of the water radiolysis, with its highly reactive intermediates •H, •OH and *e*_aq_^−^ besides the formation of the molecular and ionic products H_2_O_2_, H_2_, and H_3_O^+^.^[^^]^ The scope of the t‐butanol is to scavenge two of the reactive intermediates namely •H and •OH via hydrogen abstraction (Equation 14). The resulting t‐butanol radical is redox inert and much less reactive than •H and •OH and often reported to just recombine to pinacol.^[^ ^39^ ^]*^ (^*^ Our study indicates that POV6 {V_6_^V^} is also reacting with neutral radicals like •CH_2_(CH_3_)_2_C(OH) and •CH_3_ but the rate constants are orders of magnitude lower than the rate constants of the reduction reactions by *e*_aq_^‐^ and (CH_3_)_2_•C(OH)).

(13) $$H_{2}O{↭}^{•}\mathrm{OH}{,}^{•}H,{e_{\left(aq\right)}}^{-},H_{2},H_{2}O_{2}$$

(14)

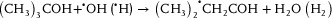

(15) $$\mathrm{POV}6\left\{{V_{6}}^{V}\right\}+{e_{\mathrm{aq}}}^{-}\to \mathrm{POV}6\left\{{V_{5}}^{V}V^{\mathrm{IV}}\right\}$$

In the second approach (CH_3_)_2_•C(OH) was utilized as a powerful reductant (−1.39 V vs NHE),^[^ ^36^ ^]^ formed in a standard procedure irradiating N_2_O saturated dilute solutions of POV6 containing 5 vol% 2‐propanol. Under such conditions *e*_aq_^−^ from the water radiolysis is quantitively converted into •OH.^[^ ^40^ ^]^ The •OH (the primary from the water radiolysis as well as the secondary from the conversion of *e*_aq_^−^ into •OH) and the •H react with (CH_3_)_2_CH(OH) under hydrogen abstraction forming (CH_3_)_2_•C(OH).^[^ ^41^ ^]^

(16) $$N_{2}O+H_{2}O+{e_{\mathrm{aq}}}^{-}\to^{•}\mathrm{OH}+OH^{-}+N_{2}$$

(17)

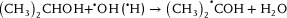

(18) $$\begin{array}{ccc} & & \mathrm{POV}6\left\{{V_{6}}^{V}\right\}+{{\left(CH_{3}\right)}_{2}}^{•}\mathrm{COH}\to \mathrm{POV}6\left\{{V_{5}}^{V}V^{\mathrm{IV}}\right\}\\ & & \;+{\left(CH_{3}\right)}_{2}\mathrm{CO}+H^{+} \end{array}$$

**Figure 4:**
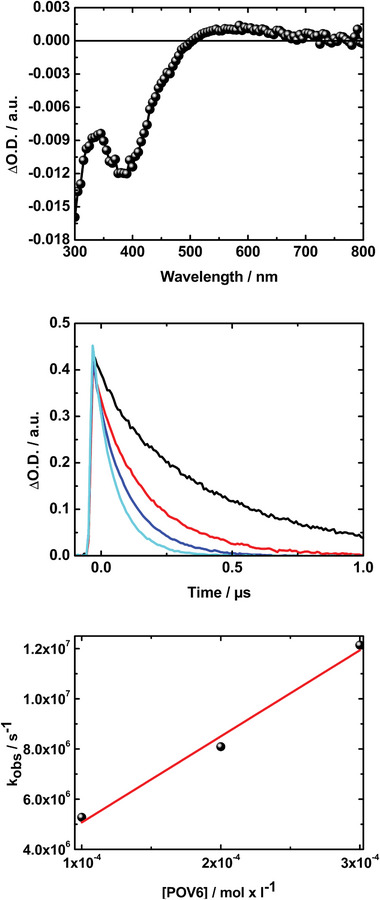
**Top**: Pulse radiolysis transient absorption spectrum of 1 × 10^−4^ m POV6 in N_2_ saturated water, containing 5 vol% *t*‐butanol – 2 µs after the electron pulse (15 ns FWHM, 85 Gy / pulse). **Middle**: Corresponding time absorption profiles at 720 nm showing the decay of the transient absorption of the solvated electron without {V_6_^V^} (black), and with {V_6_^V^} concentrations of 1 × 10^−4^ m (red), 2 × 10^−4^ m (blue), and 3 × 10^−4^ m (cyan). **Bottom**: Corresponding plot of the pseudo‐first‐order rate constant for the reaction of POV6 with *e*_aq_^−^ versus POV6 concentration.

When *e*_aq_^−^ was employed as reductant immediately after the electron pulse, the characteristic broad transient absorption of the *e*_aq_^−^ with its characteristic maximum ≈720 nm was discernable. This decays rapidly in the presence of POV6, giving rise to a transient bleaching (negative transient absorption) in the UV region of the optical spectrum with a minimum ≈370 nm, where POV6 exhibits ground state absorption, and a broad weak transient absorption band in the visible range of the optical spectrum maximizing ≈550 nm. Both features of the transient absorption spectrum are attributed to the formation of one‐electron reduced POV6 {V^V^_5_V^IV^} (**Figure** **4**). The rate constant for the one‐electron reduction of POV6 by *e*_aq_^−^ was determined from the decay of the 720 nm transient absorption of *e*_aq_^−^ at various POV6 concentrations assuming a pseudo‐first order kinetic giving a second order rate constant of 3 × 10^10^ L × mol^−1^ × s^−1^ (Figure 4).

Similarly, when the one‐electron reduction of {V_6_^V^} by (CH_3_)_2_•C(OH) was probed immediately after the electron pulse, the transient absorption of (CH_3_)_2_•C(OH) is discernable. This decays rapidly and gives rise to a transient bleaching in the UV region ≈370 nm (see Figure S10, Supporting Information) and a weak transient absorption maximizing ≈550 nm, which is similar to the transient absorption spectrum observed, when POV6 was reduced by *e*_aq_^−^. The formation of one‐electron reduced POV6 {V^V^_5_V^IV^} species is therefore anticipated. It should be noted that under our experimental conditions no further transformations of the POV6 {V^V^_5_V^IV^} is observed within the upper detection time limit of our pulse radiolysis setup (350 µs).

The rate constant for the reduction of POV6 by (CH_3_)_2_•C(OH) was obtained by analyzing the formation of the transient bleaching ≈370 nm assuming a pseudo first order kinetic. A bimolecular rate constant of 2.3 × 10^8^ L × mol^−1^ × s^−1^ was revealed. But taking a closer look at the plot of the pseudo first order rate constant versus the POV6 concentration, a substantial intercept is observed indicating a more complex reaction mechanism. One can speculate if the intercept may relate to an equilibrium between POV6 and (CH_3_)_2_•C(OH) and the formation of an [{V_6_}‐(CH_3_)_2_•C(OH)] intermediate complex prior to the electron transfer forming POV6 {V^V^_5_V^IV^}.

With the transient absorptions of the one‐electron reduced POV6 on hand, nanosecond laser photolysis experiments exciting solutions of POV6 in acetonitrile and water with 355 nm laser pulses were conducted to study the excited states of POV6. In nitrogen‐saturated acetonitrile, a weak transient absorption spectrum was observed with a minimum in the UV ≈350 nm and a very weak, insignificant transient absorption in the visible region of the spectrum, which persisted on a microsecond time scale. But due to the weakness of the signal and because of this very limited signal‐to‐noise ratio, we refrain from giving an exact lifetime (see Figure S11, Supporting Information). Due to its microsecond long lifetime and the fact that we lacked any transient absorption signals when measuring in oxygen saturated solutions, we assume that this transient absorption is related to the first triplet state of POV6. In aqueous solutions we were unable to measure a transient absorption spectrum with our nanosecond laser photolysis transient absorption setup. The fact that we were not able to obtain a nanosecond transient absorption spectrum in aqueous POV6 solutions triggered the notion that photoexcited POV6 may react with water or its ionic products. Theoretical calculations (vide supra) have shown that the oxidation of neutral water is energetically not feasible, neither from the first triplet state of POV6 nor from the first excited singlet state. However, the same theoretical investigation revealed that the oxidation of OH^–^ to •OH is energetically feasible from both the first excited singlet state as well as from the first triplet state. Such photoinduced oxidation capabilities were previously reported for some POM systems.^[^ ^42^, ^43^ ^]^

To corroborate the notion that photoexcited POV6 may oxidize OH^–^ to •OH, solutions containing 1 × 10^−4^ m POV6 and 1 × 10^−4^ m coumarin or disodium terephthalate were illuminated with a 150 W XBO lamp, respectively. The fluorescence of the solution was measured at different illumination times. Coumarin is a well‐established sensor for •OH in photocatalysis since •OH reacts with coumarin forming among other hydroxy adducts 7‐hydroxy‐coumarine, which other than pristine coumarin exhibit upon photoexcitation a strong fluorescence with a maximum ≈455 nm in water.^[^ ^44^, ^45^ ^]^ Also disodium terephthalate is an established fluorescence sensor for •OH radicals since it reacts with •OH to 2‐hydroxy‐terephthalate, which exhibits a strong fluorescence spectrum with a maximum at 425 nm.^[^ ^46^ ^]^

When aqueous solutions of 1 × 10^−4^ m POV6 and 1 × 10^−4^ m coumarin were illuminated, an increase of the 7‐hydroxy‐coumarine centered fluorescence with increasing illumination time was observed indicating toward the formation of •OH radicals (see Figure S12, Supporting Information). Additional evidence for •OH formation was obtained from illumination and subsequent fluorescence studies of an aqueous solution of 1 × 10^−4^ m POV6 and 1 × 10^−4^ m disodium terephthalate, showing an increase in 2‐hydroxy‐terephthalate centered fluorescence with increasing irradiation time (see Figure S13, Supporting Information), corroborating the formation of •OH, so that based on photochemical and radiation chemical studies it is safe to conclude that the photoexcited states of POV6 are a strong oxidizing agent capable of oxidizing OH^−^ to •OH, which may than recombine into H_2_O_2_.

### 2.4 XPS Studies

The proposed electronic communication between two POV6 complexes could also be demonstrated by XPS studies of the solid‐state sample irradiated by soft X‐rays.

**Figure 5:**
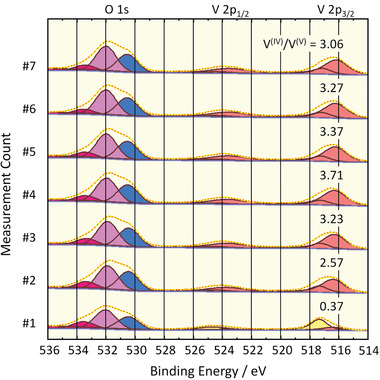
XPS measurement series of the O 1s and V 2p core‐levels to analyze the stability of the POV6 compound under X‐ray irradiation for repeated measurements at one sample position.

Vanadium compounds are known to degrade under X‐ray irradiation.^[^ ^47^ ^]^ To investigate the effect of X‐rays on the title POV6 compound, a sample of it was measured at one position seven times at 1 h intervals over the entire time with the X‐ray source activated (see the Supporting Information). The V^IV^/V^V^ ratio^[^ ^48^ ^]^ is found to first increase with each XPS measurement (**Figure** **5**), indicating accumulation of the X‐ray effect on POV6. The reason for this can be the formation of material damage, POV6 charge state activation, or POV6 product formation. After 2 h (third measurement) the V^IV^/V^V^ ratio saturates at a value >3. The reason for the accumulation effect cannot be determined based on the investigations. However, in subsequent studies, care was taken to keep the X‐ray exposure very short to avoid POV6 changes, and the possible X‐ray effect was taken into account when choosing the experimental design.

The influence of the electron flood gun for charge neutralization on the POV6 was also studied. Thick POV6 layers demonstrate considerable charging, thus requiring the use of the flood gun. By contrast, very thin POV6 layers exhibit only negligible charging effects, allowing them to be measured without the flood gun. The measurements depicted in Figure 5 were performed without the flood gun. However, a time series with the flood gun yielded equal results, so the electron irradiation effect on the POV6 can be ruled out. In the following experiments, the flood gun was used.

To understand the effect of UV irradiation (with *λ* = 185 nm, see the SI for details) two measurement scenarios were explored: 1) Fresh POV6 samples were measured before and after UV irradiation at the same positions to gain insight into the influence of local and processing‐based variations on the vanadium oxidation state ratio. 2) Fresh POV6 samples were measured only after UV irradiation to obtain an understanding of the V^IV^/V^V^ ratio without any prior material degradation caused by X‐rays.

**Figure 6:**
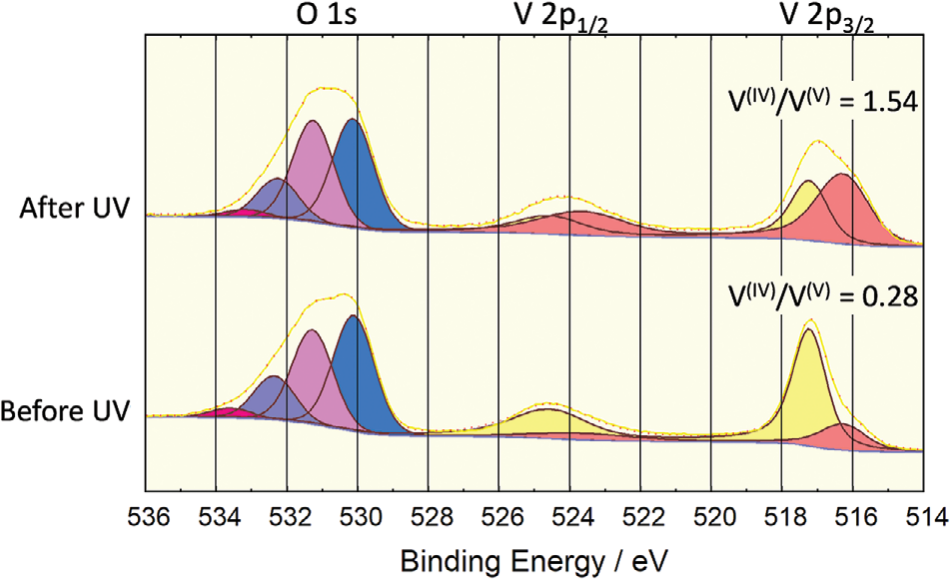
XPS measurements of the O 1s and V 2p core‐levels before and after 10 min UV exposure at the same sample position.

Scenario 1 is depicted in **Figure** **6**. The V^IV^/V^V^ ratio of POV6 samples without UV irradiation was in the range of 0.2–0.4. After UV irradiation, the V^IV^/V^V^ ratio increases significantly to 0.9–1.8. The width of the ranges is assumed to be due to structural material parameters such as layer thickness and surface roughness of the POV6 layer, which can vary locally and from sample to sample as a result of the spin‐on technique. Samples measured only after UV irradiation (scenario 2) showed the same V^IV^/V^V^ ratio range of 1.4–1.7 as the previous XPS measurement. The UV‐induced effect of the POV6 is obviously much stronger than the effect of X‐ray irradiation during a single XPS measurement. The measured result supports the elaborated consideration of photoproduct formation with reduced vanadium oxidation state.

Furthermore, the temporal stability of the POV6 photoproduct was investigated. For this reason, the POV6 samples were first exposed to UV irradiation to reduce the POV6. Then, after a well‐defined pause XPS was measured. Three different pause times of 1, 6, and 24 h were tested. In two series of measurements, one with scenario 1 and one with scenario 2, the V^IV^/V^V^ ratio was found to be in the range of 1.4–1.7 without any correlative effect of pause time. This result indicates a POV6 photoproduct stable over several hours.

## 3 Conclusion

Our combined experimental and computational studies reveal that POV6 is an excellent electron acceptor upon photoexcitation. Pulse radiolysis, quantum chemistry calculations, and solution photochemistry demonstrate that POV6 is capable of oxidizing OH^−^ to ●OH in water solution. The formation of reduced vanadium(IV) centers in a sample of photochemically induced POV6 powder are due to the oxidation of OH^−^ from air moisture on the surface of the POV6 powder to ^●^OH or to electron exchange between two POV6 complexes. Proton transfer reactions from acetonitrile or the tetrabutylammonium cation to the T_1_ state of POV6 in acetonitrile solution could be excluded due to the high activation barrier. Thus, the reduced species POV6 {V^V^_5_V^IV^} is mainly generated by oxidation of a second POV6 in water‐free environments of this study. The obtained results have great potential for the development of biomimetic physical processes, programming of complex chemical matter, and continuous in‐memory processing powered by electrical energy and light.

## Conflict of Interest

The authors declare no conflict of interest.

## Supporting information

Supporting Information

## Data Availability

The data that support the findings of this study are available in the supplementary material of this article.
