# Supplementary material for: Reversible Optical Switching of Polyoxovanadates and Their Communication via Photoexcited States
Source: Adv Sci (Weinh). 2024 Jun 13;11(30):2401595. doi: 10.1002/advs.202401595 (PMC11321688; doi:10.1002/advs.202401595)
Supplement: Supplementary file 1 — Supporting Information [file ADVS-11-2401595-s001.docx]

**Supporting Information**

**Reversible Optical Switching of Polyoxovanadates and Their Communication via Photoexcited States**

Eric Vogelsberg,^[a]^ Jan Griebel,^[a]^ Iryna Engelmann,^[a]^ Jens Bauer,^[a]^ Florian Taube,^[b]^  Björn Corzilius,^[b,c]^ Stefan Zahn,^[a]^* Axel Kahnt,^[a]^* and Kirill Yu. Monakhov^[a]^*

^[a]^ Leibniz Institute of Surface Engineering (IOM), Permoserstr. 15, 04318 Leipzig, Germany

^[b]^ Institute of Chemistry and Department of Life, Light & Matter, University of Rostock, Albert-Einstein-Str. 25-27, 18059 Rostock, Germany

^[c]^ Leibniz-Institute of Catalysis (LIKAT), Albert-Einstein-Str. 29a, 18059 Rostock, Germany

E-mails: stefan.zahn@iom-leipzig.de, axel.kahnt@iom-leipzig.de, kirill.monakhov@iom-leipzig.de

**CONTENT**

1. Experimental details
2. NMR spectra
3. UV/vis spectra
4. DFT and TD-DFT calculations
5. Radiation chemical and photochemical spectra
6. XPS data
7. References
8. **Experimental details**

**EPR measurement**

The EPR spectra were measured with a X-band EPR spectrometer (ELEXSYS, BRUKER) with a cavity operating in the TE_102_ mode at rt. The spectrometer setup was a microwave power 20 mW, a receiver gain of 3ꞏ10^4^, and a modulation amplitude of 0.3 mT. The simulation was performed using the software tool Easyspin.^[1]^

**MAS-NMR measurement**

The solid-state ^51^V-NMR experiments were carried out on a Bruker AVANCE III HD spectrometer operating at 400.5 MHz proton frequency with a Bruker ASCEND DNP 9.4 T widebore (89 mm) magnet and using a Bruker 4 mm MAS WVT HXY probe. Experimental parameters were set in accordance with earlier studies.^[2]^ The solid echo experiment was performed at an MAS frequency of 15 kHz with a pulse separation of one rotor period (66.7 µs), pulse lengths of 1 µs and flip angles of approximately 30°. Detection was performed with a spectral width of 1.25 MHz.

**MAS-NMR simulation and fitting**

Simulations of ssNMR spectra of V_6_O_19_(N_3_) were performed using the ssNake NMR processing and fitting software (v1.4).^[3]^ The FIDs underwent digital filter correction, Fourier transformation, indirect referencing to adamantane (37.8 ppm, 28.7 ppm), baseline correction, and zeroth and first-order phase correction. Fitting was carried out on the data obtained at 15 kHz MAS frequency using the chemical shift anisotropy (CSA) and quadrupolar fitting function. The Haeberlen convention (*δ*_iso_ - *δ*_aniso_ - *η*_CSA_) was used to specify CSA, while *C*_q_ and *η*_NQI_ were utilized to describe the quadrupolar interaction strength. These five parameters, along with integral, Lorentz line broadening, and Gauss line broadening parameters, were kept variable for fitting. Other fixed fitting parameters included *I* = 7/2, the Cheng number (number of powder averages) of 15, *ω*_MAS_ = 15 kHz, and calculation of 128 rotational sidebands was allowed. Euler angel *α*, ⁠*β* and *γ* were set to 0°. To validate the fitted parameters, spectra measured at 12.5 and 10 kHz MAS frequencies were simulated using the settings mentioned before together with the parameters obtained from the spectrum measured at 15 kHz MAS frequency only varying the MAS frequency. This process was completed without any issues. The final values are as follows: *δ*_iso_ = –515 ppm, *δ*_aniso_ = –415 ppm, *η*_CSA_ = 0.06, *C*_q_ = 5.28 MHz, *η*_NQI_ = 0.416, integral = 3.41ꞏ10^4^, Lorentzian broadening = 195 Hz, Gaussian broadening = 19.9 ppm. Note that all three spectra were recorded with the same experimental settings described previously.

1. **NMR spectra**

**
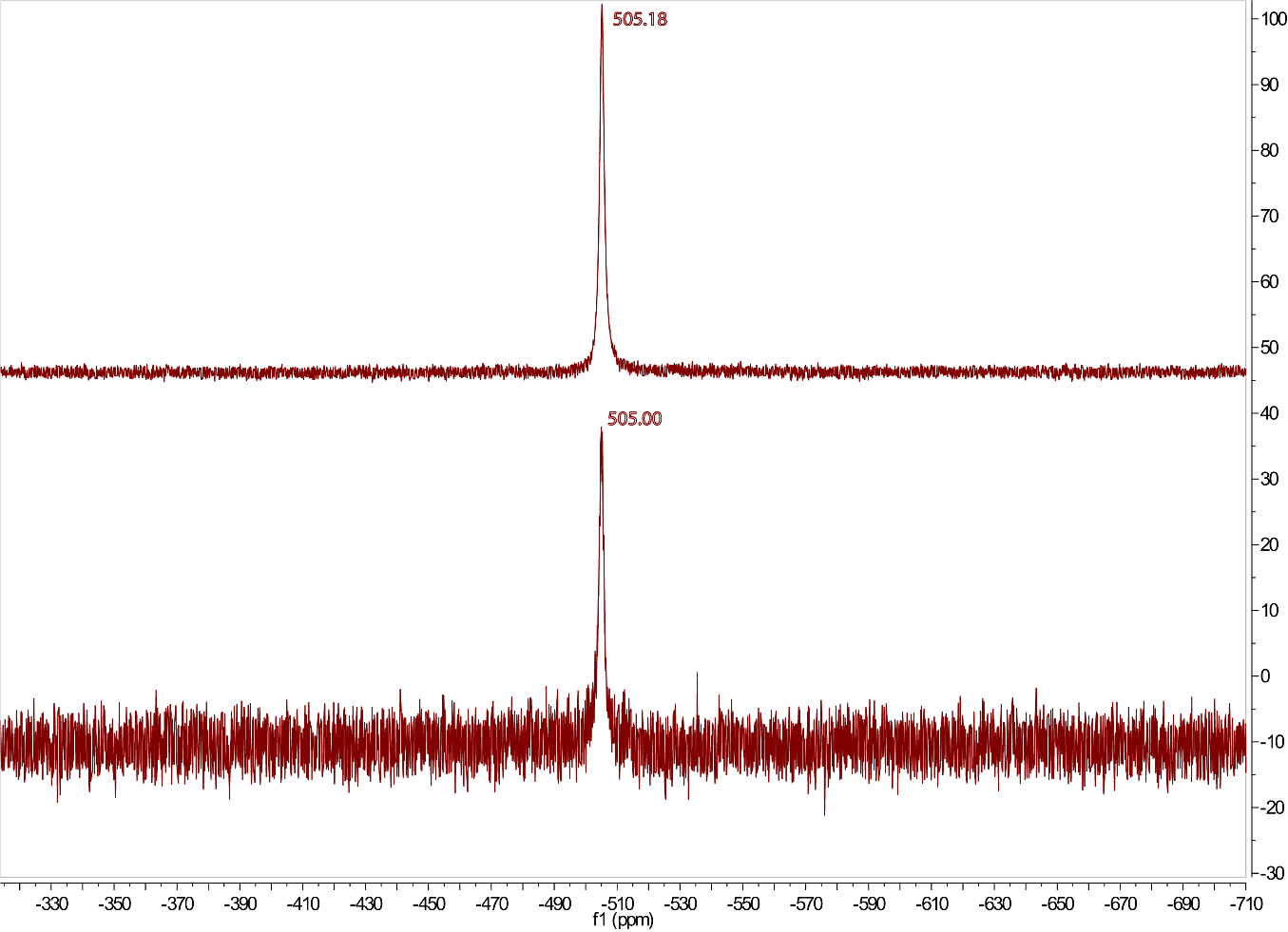
**

**Figure S1.** **Top**: ^51^V-NMR of (nBu_4_N)_2_[V_6_O_13_((OCH_2_)_3_CCH_2_N_3_)_2_] in CD_3_CN solution after irradiation. **Bottom**: ^51^V-NMR of (nBu_4_N)_2_[V_6_O_13_((OCH_2_)_3_CCH_2_N_3_)_2_] in CD_3_CN solution after irradiation.


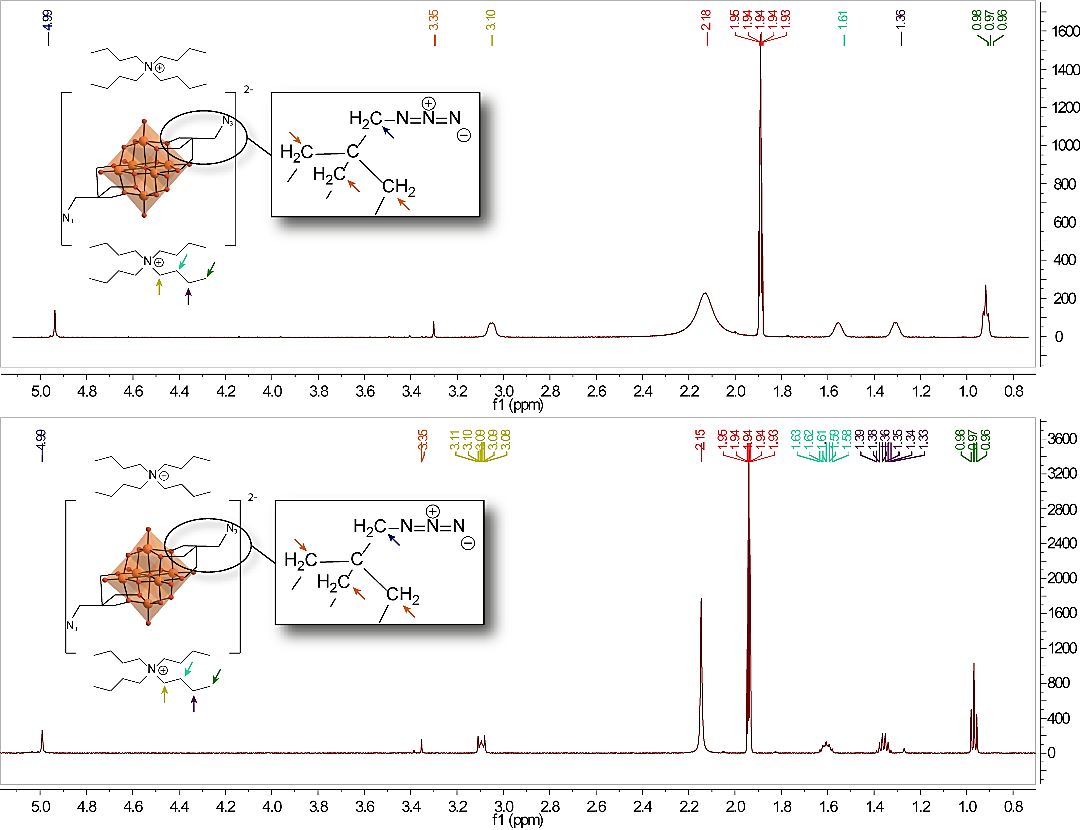


**Figure S2. Top**: ^1^H-NMR of (nBu_4_N)_2_[V_6_O_13_((OCH_2_)_3_CCH_2_N_3_)_2_] in CD_3_CN solution after irradiation. **Bottom**: ^1^H-NMR of (nBu_4_N)_2_[V_6_O_13_((OCH_2_)_3_CCH_2_N_3_)_2_] in CD_3_CN solution before irradiation. No changes in the multiplicity of the signal and the chemical shift are observed. The only change is the broadening of the signal caused by the charge-spin coupling of the newly generated unpaired electron.


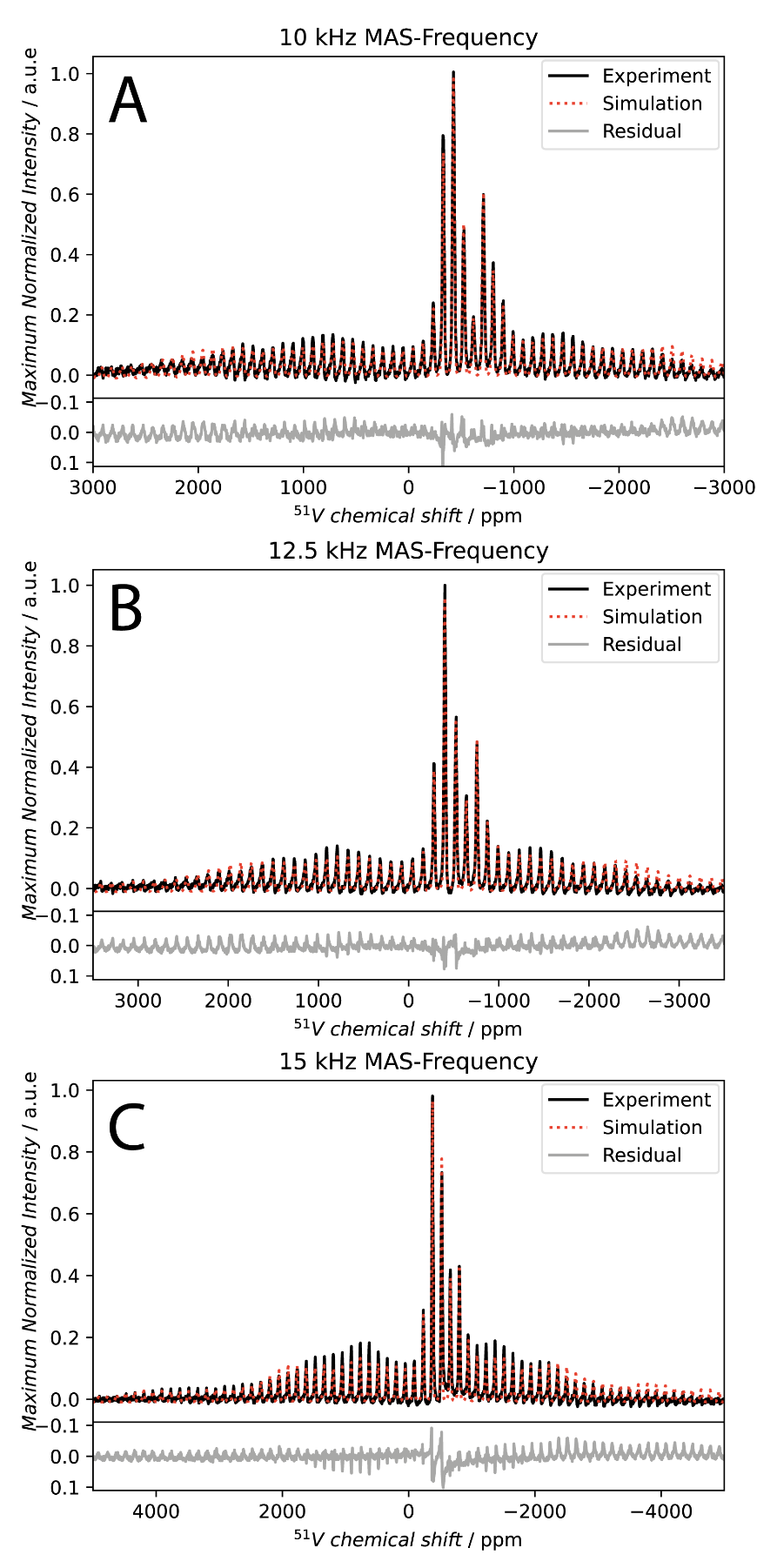


**Figure S3.** Experimental and simulated data of the MAS ssNMR experiment performed with dried (nBu_4_N)_2_[V_6_O_13_((OCH_2_)_3_CCH_2_N_3_)_2_]. The experimental data obtained at 15 kHz (C), were used to create a fit, that was validated with the experimental data measured at 12.5 kHz and 10 kHz(A,B).

1. **UV/vis spectra**


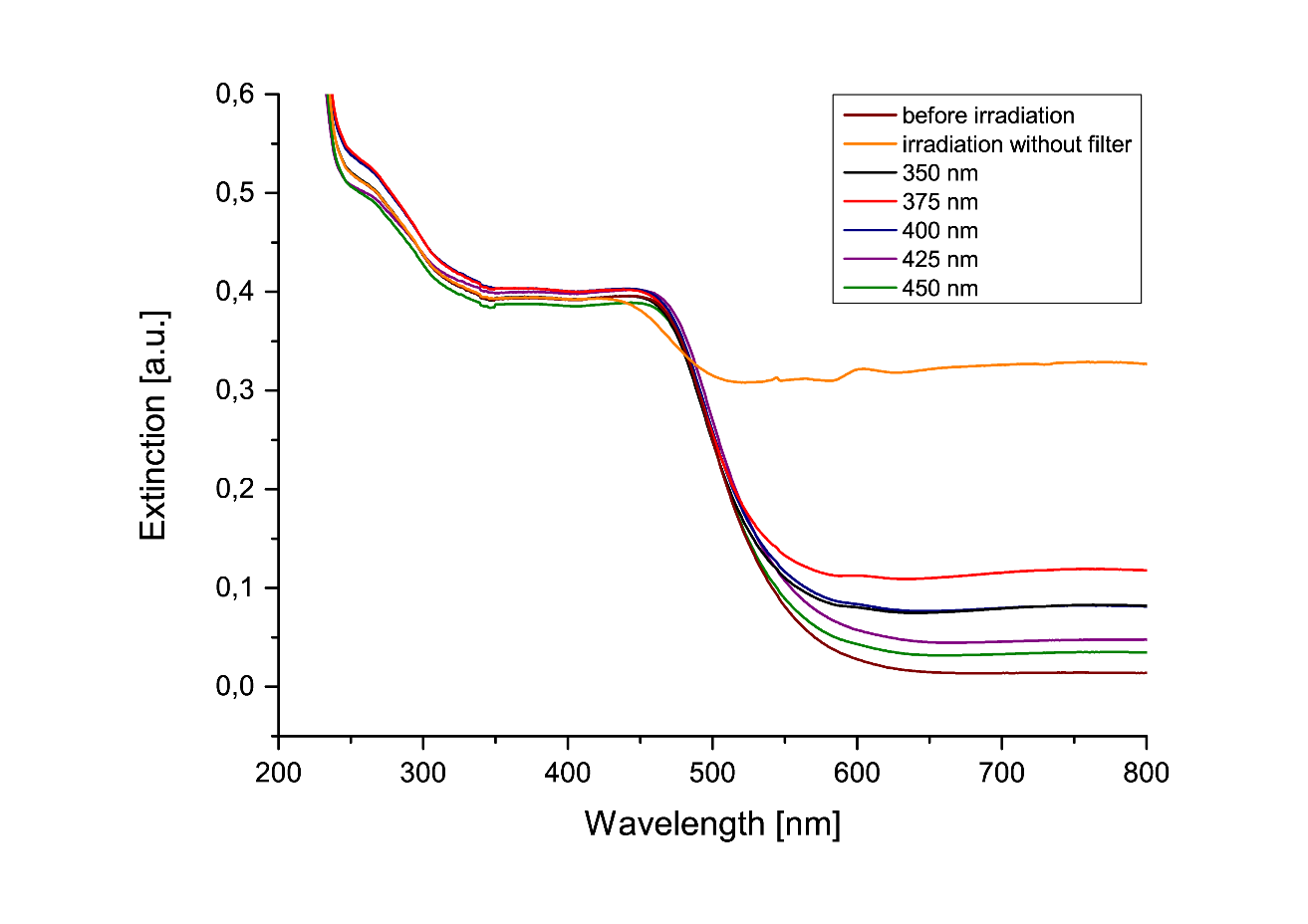


**Figure S4.** UV/vis absorption spectra of (nBu_4_N)_2_[V_6_O_13_((OCH_2_)_3_CCH_2_N_3_)_2_] in MeCN solution. All samples were irradiated for 90 s at the same distance with a Hg lamp. Different wavelengths were realized by placing a filter that allowed only to pass the desired wavelength between the sample and the lamp.


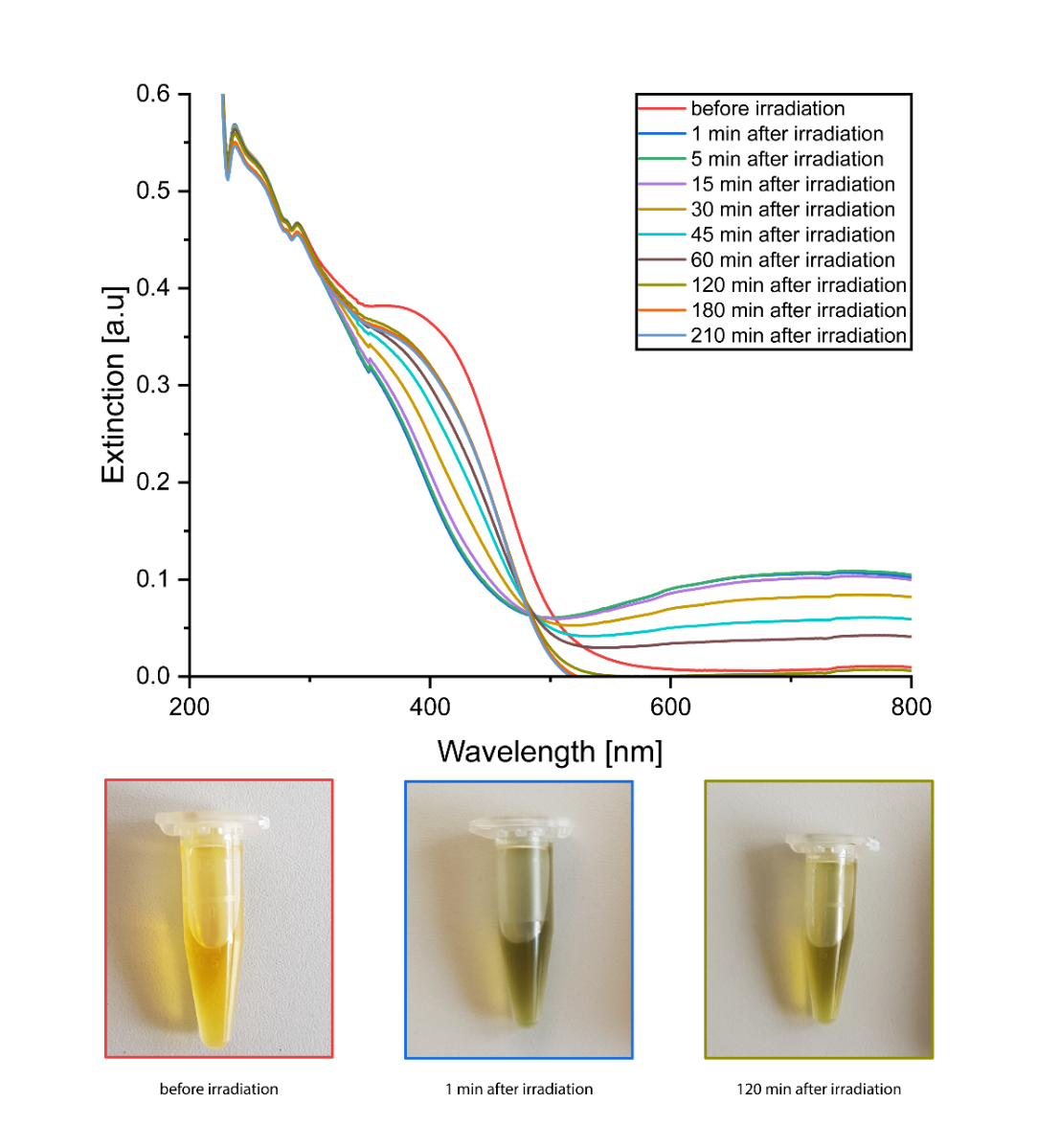


**Figure S5.** UV/vis absorption spectra of (nBu_4_N)_2_[V_6_O_13_((OCH_2_)_3_CCH_2_N_3_)_2_] in MeCN solution. The spectra are taken from the same sample before and after the 90 s irradiation with a 365 nm LED.


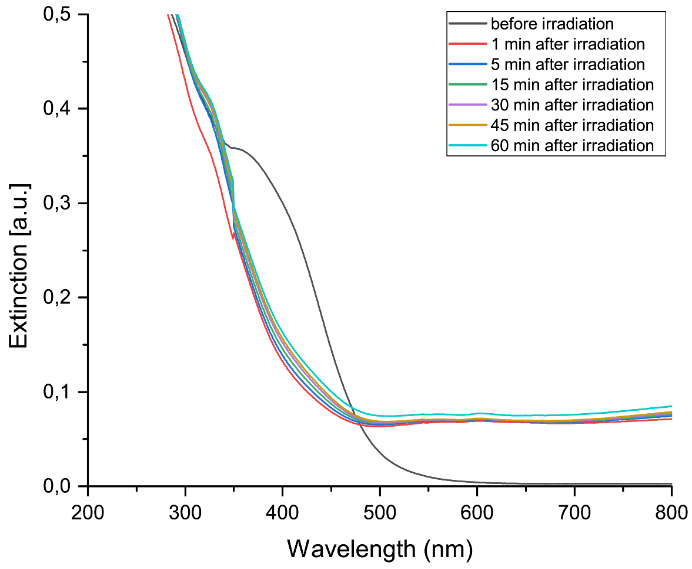

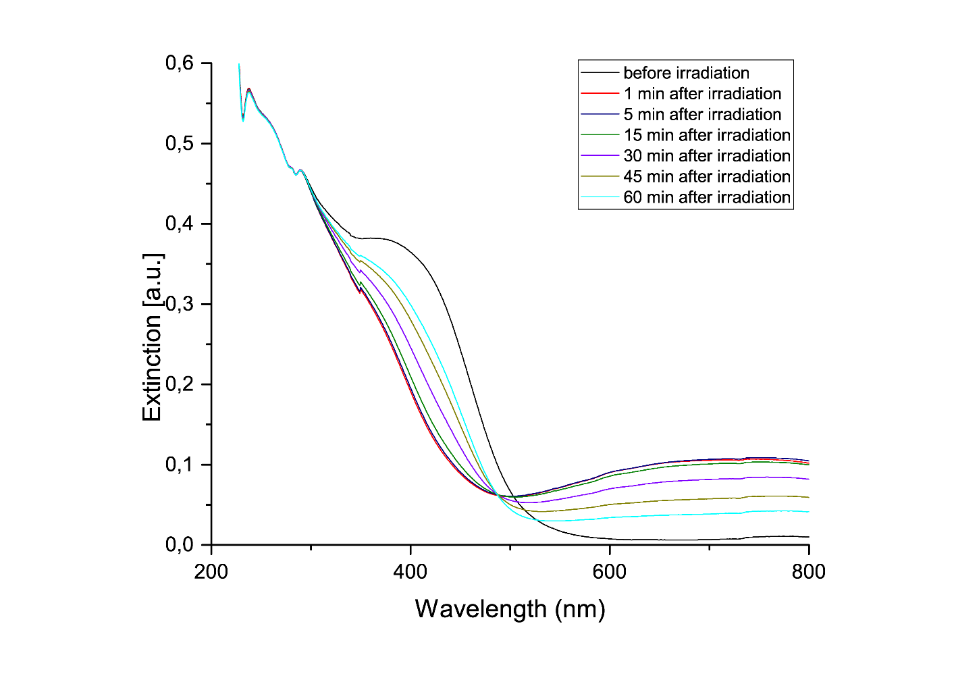


**Figure S6.** **Left**: UV/vis absorption spectra of (nBu_4_N)_2_[V_6_O_13_((OCH_2_)_3_CCH_2_N_3_)_2_] in 100%-vol MeCN solution following a 60 min period after the irradiation. **Right**: Spectra of (nBu_4_N)_2_[V_6_O_13_((OCH_2_)_3_CCH_2_N_3_)_2_] in 20%-vol MeCN solution following a 60 min period after the irradiation.


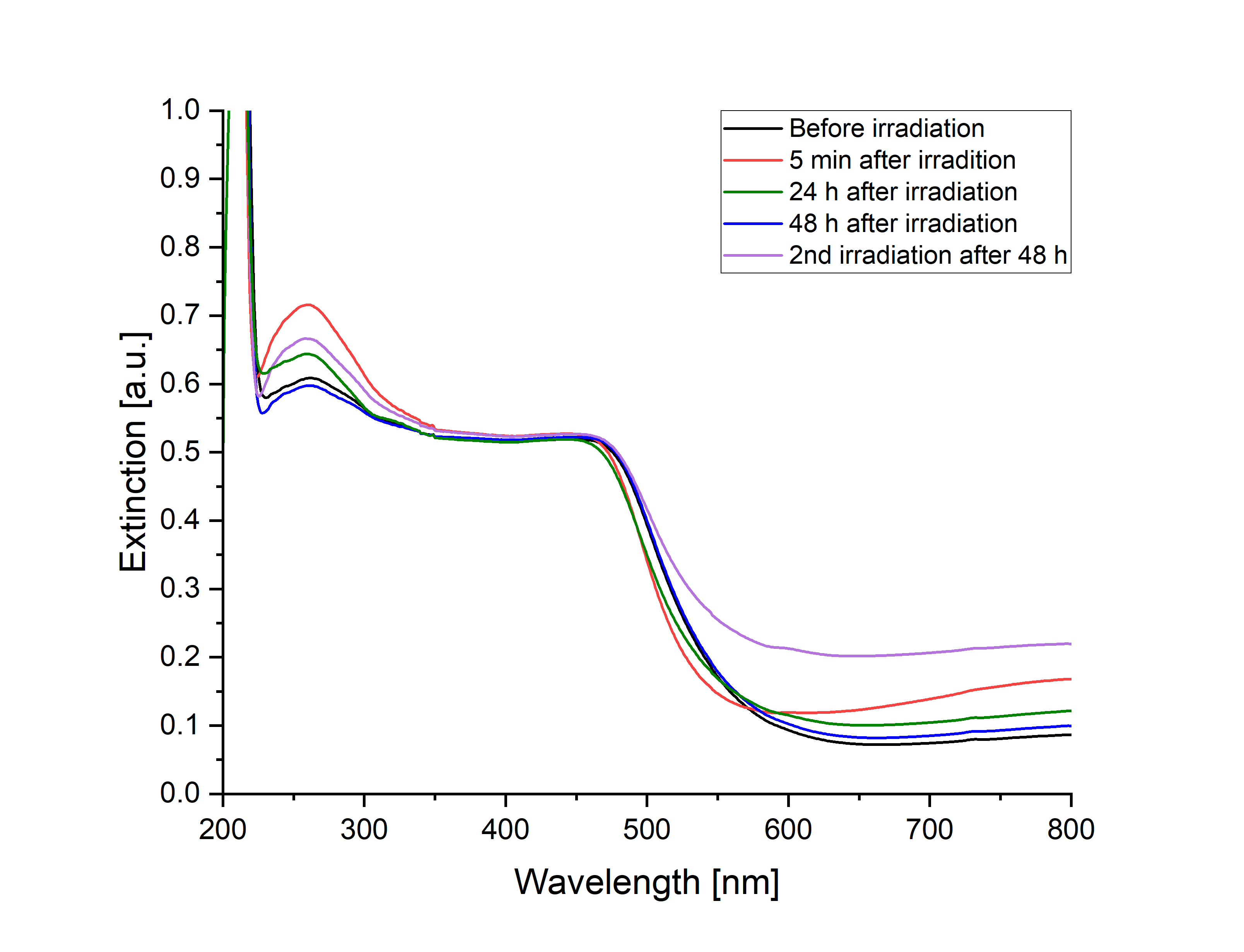

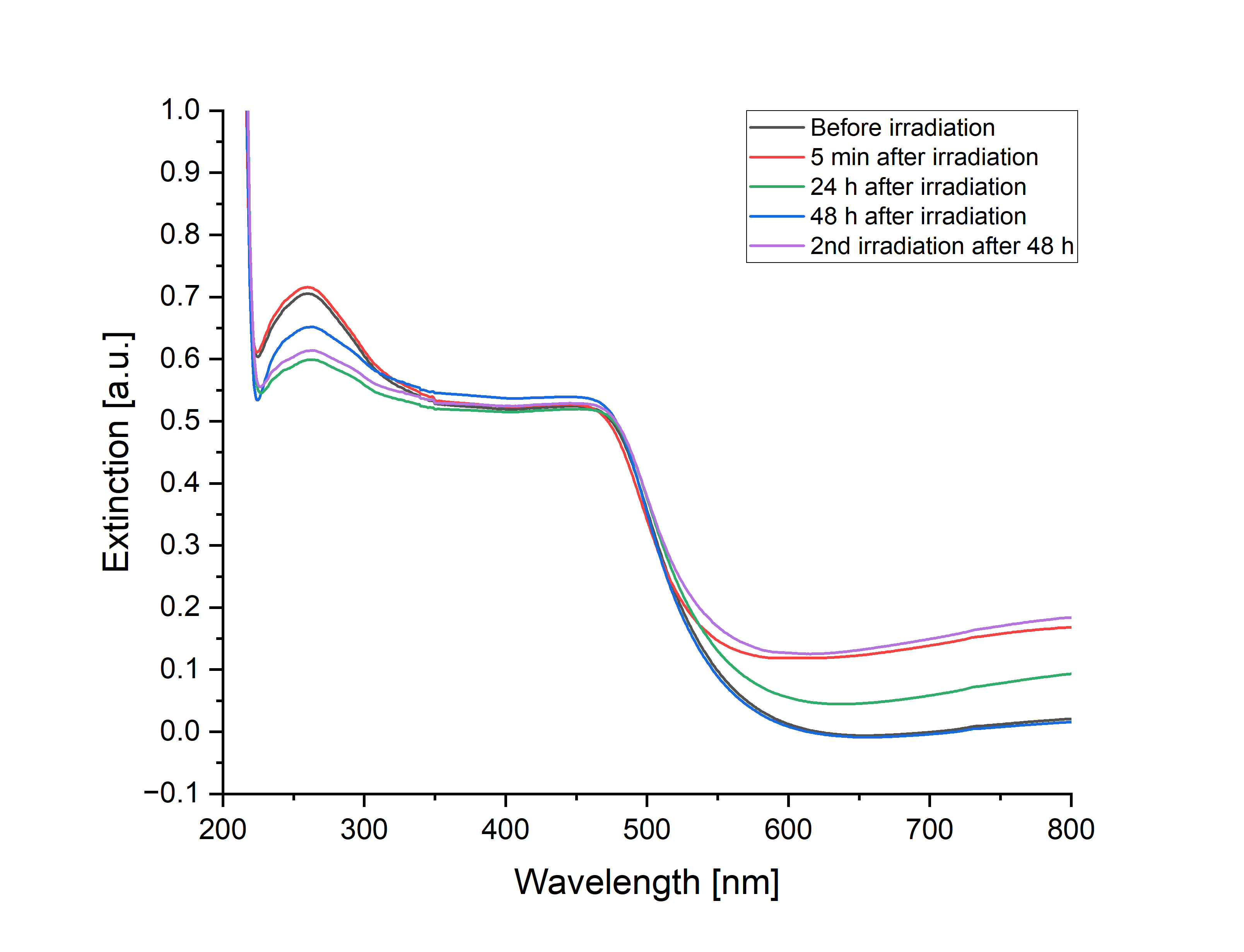


**Figure** **S7. Left**: UV/vis absorption spectra of (nBu_4_N)_2_[V_6_O_13_((OCH_2_)_3_CCH_2_N_3_)_2_] in 100%-vol MeCN over a period of 48 h, following the initial irradiation procedure. **Right**: UV/vis absorption spectra of (nBu_4_N)_2_[V_6_O_13_((OCH_2_)_3_CCH_2_N_3_)_2_] in 80%-vol MeCN over a period of 48 h, following the initial irradiation procedure. In both samples, a second irradiation was performed after 48 h to see if the initial change in the absorption spectrum could be reproduced. The irradiation was performed for 90s at the same distance with a 365 nm UV LED.

1. **DFT and TD-DFT calculations**

The ORCA program package was employed for all quantum chemical calculations.^[4]^ All structures were optimized with the revised def2-TZVP basis set^[5]^ and a modified B3LYP functional where the Hartree-Fock exchange was reduced to 15%. This was recommended by Reiher and coworkers to estimate the energy splitting between different spin states of ground state structures.^[6]^ Minima on the potential energy surface were always confirmed by semi-numerical frequency calculations. Solvation effects of acetonitrile or water were considered by the conductor-like polarizable continuum model (C-PCM) during structure optimization and frequency calculations.^[7]^ Subsequently to structure optimization, a single point energy calculation was carried out to obtain the final energy for which the def2-TZVP basis set, the revised B3LYP functional with 15% Hartree-Fock exchange and the SMD solvation model^[8]^ were employed. The energy levels of the excited states of the **S_0_** ground state geometry were determined with the def2-TZVP basis set in combination with the range-separated CAM-B3LYP functional employing time-dependent density functional theory (TD-DFT) calculations. This functional significantly reduce the error of TD-DFT for charge transfer excitations.^[9]^ All calculations were accelerated employing the resolution of identity approximation^[10]^ in combination with the chain-of-spheres algorithm.^[11]^ The energy of the solvated electron (Δ_hyd_*G* = –151.9 kJ mol^–1^) was taken from the literature.^[12]^

POV6H {V_5_^V^V^IV^} possess three different tautomers, see Figure S6. Reported values in the publication refer always to the most stable tautomer. Furthermore, four different tautomers are possible for •N_4444_^+^, see Figure S7. Solely values for the most stable tautomer are reported in the publication.


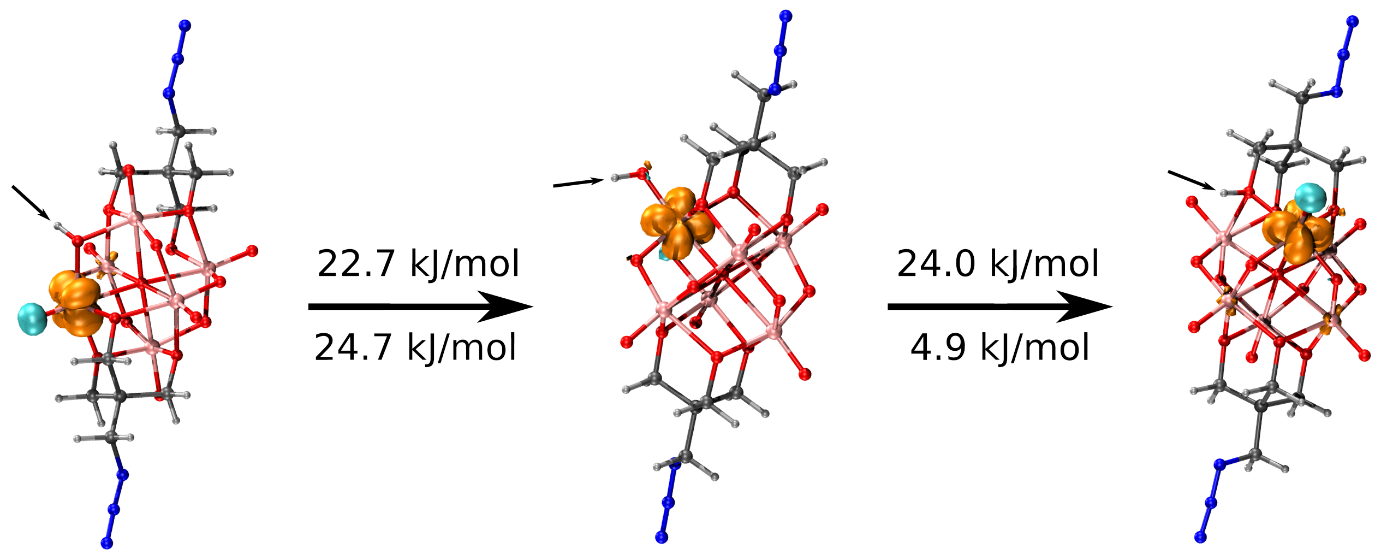


**Figure S8.** Comparison of tautomers of POV_6_H {V_5_^V^V^IV^}. The value above the arrow is the free reaction enthalpy in acetonitrile, while the value below is in water. The small arrow highlights which proton position differs in each structure. Regions of increased α and β spin density of ground state structures are colored orange and cyan, respectively.


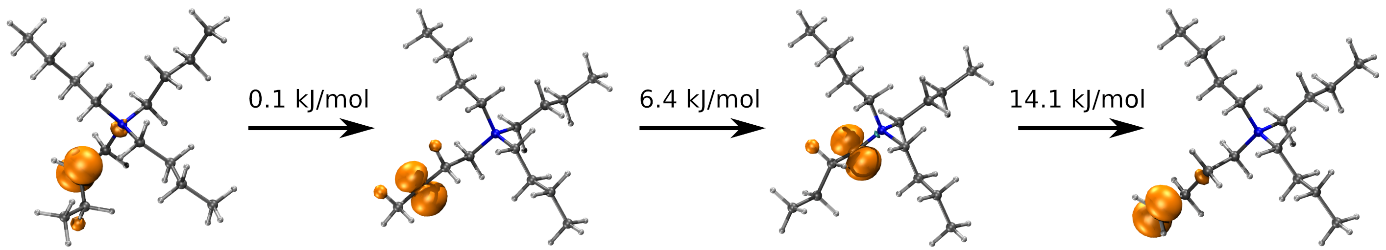


**Figure S9.** Comparison of isomers of •N_4444_^+^ in acetonitrile solution. Regions of increased α and β spin density of ground state structures are colored orange and cyan, respectively.

1. **Radiation chemical and photochemical spectra**


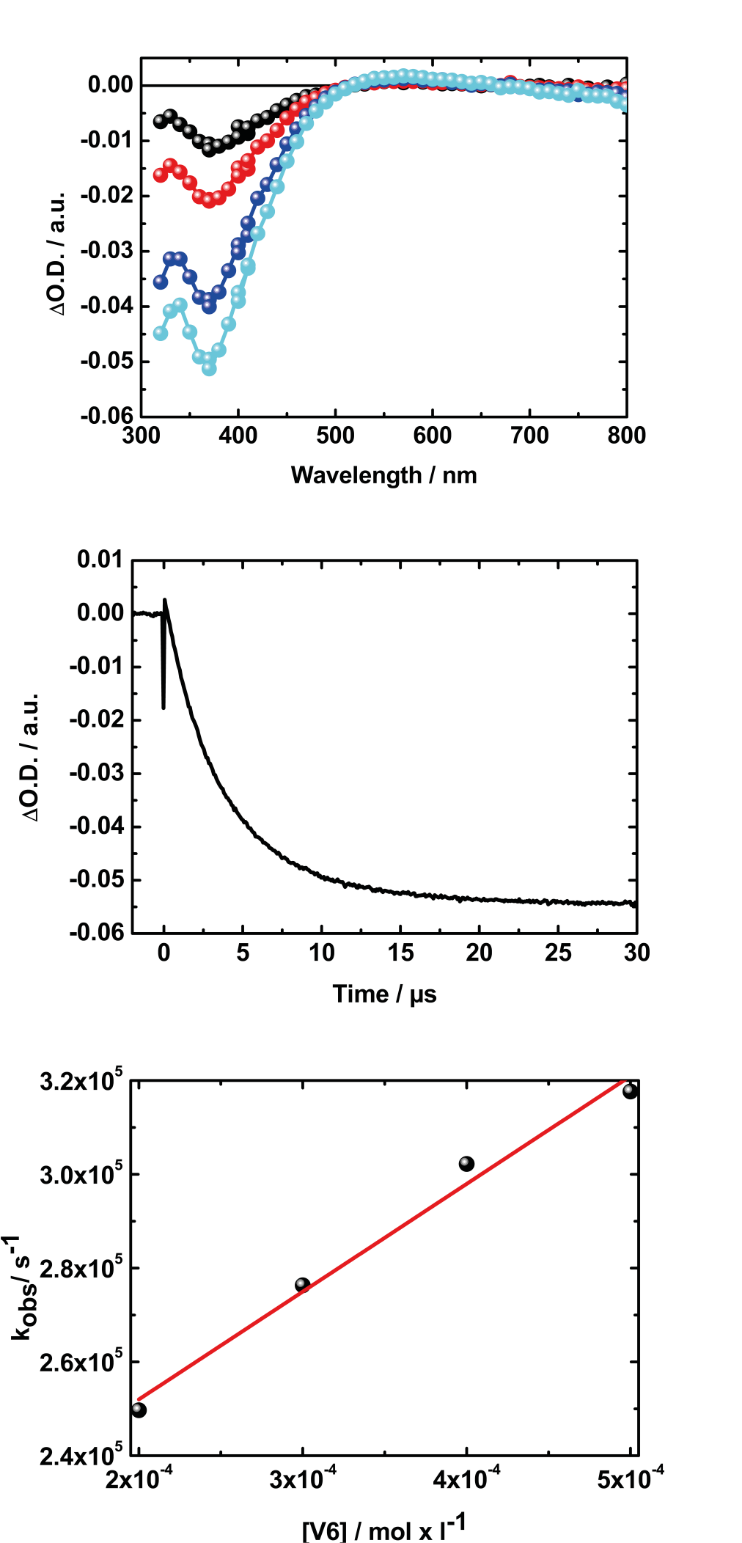


**Figure S10. Top**: Pulse radiolysis transient absorption spectra of 2 × 10^–4^ mol × L^–1^ POV6 in N_2_O saturated water, containing 5 vol% 2-propanol - 1 µs (black), 2 µs (red), 5 µs (blue), and 10 µs (cyan) after the electron pulse (15 ns FWHM, 85 Gy / pulse). **Middle**: Corresponding time absorption profiles at 370 nm. **Bottom:** Plot of the pseudo-first-order rate constant for the reaction of POV6 with (CH_3_)_2_•C(OH) versus POV6 concentration.


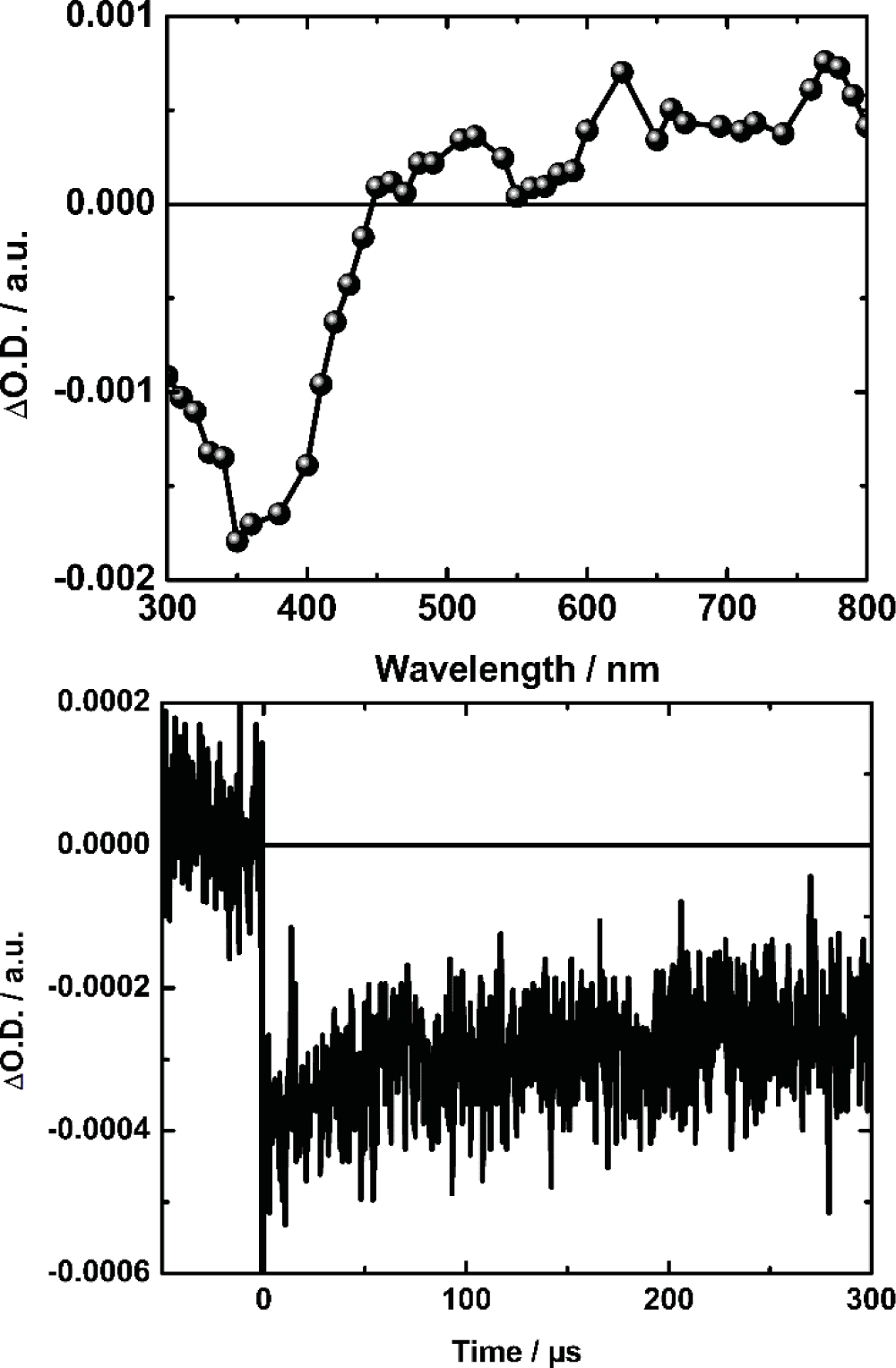


**Figure S11. Top**: Nanosecond transient absorption spectrum of POV6 in N_2_ saturated acetonitrile upon photoexcitation at 355 nm (5 ns FWHM, 5 mJ / pulse) 15 µs after the laser pulse. **Bottom**: Corresponding time absorption profiles at 350 nm.





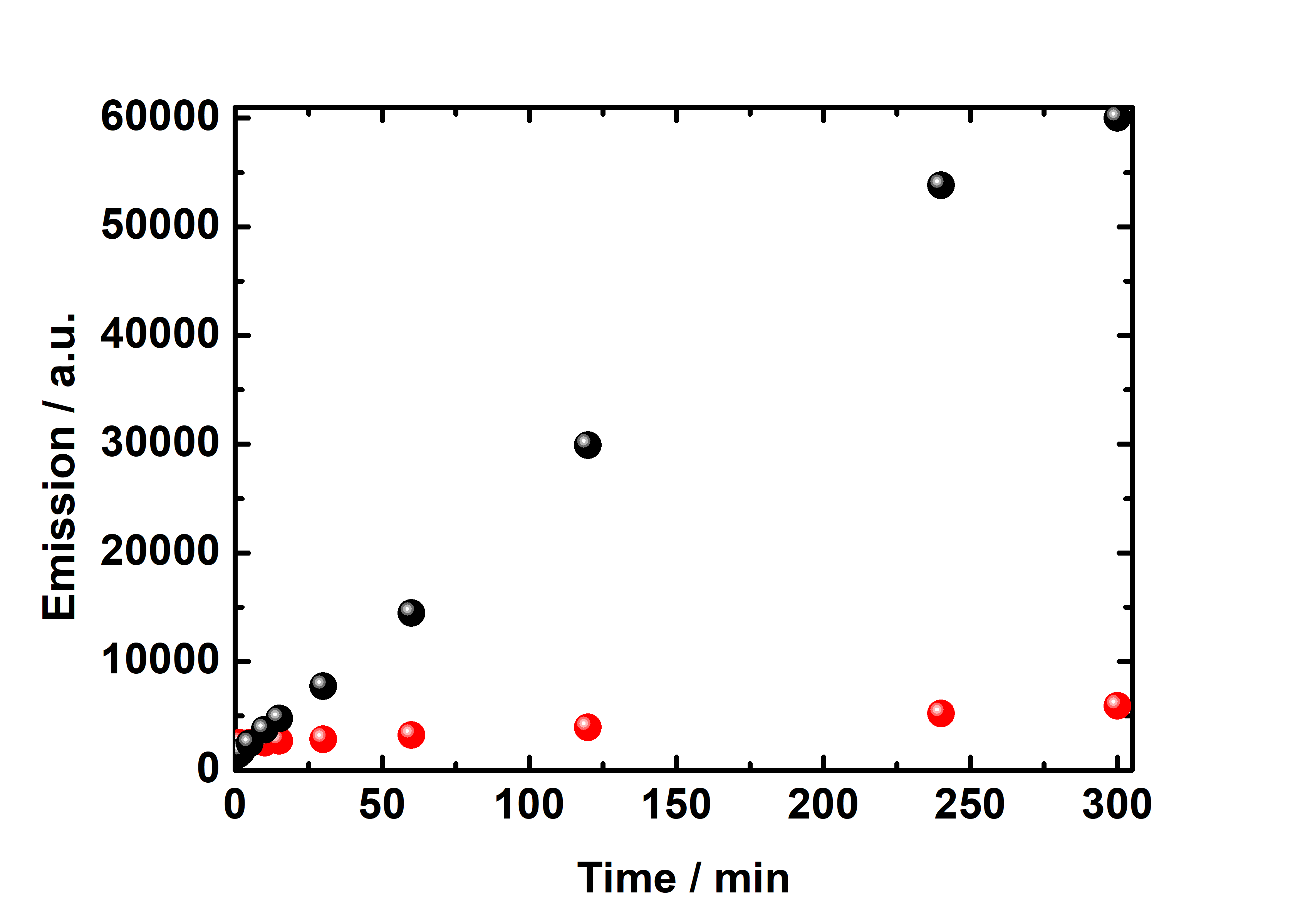


**Figure S12. Top**: Fluorescence spectra upon photoexcitation at 310 nm of POV6 (1 × 10^–4^ M) and coumarin (1 × 10^–4^ M) in water measured after different illumination times. **Bottom**: Corresponding fluorescence intensity *vs.* illumination time profiles at 455 nm (black dots). The red dots represent the fluorescence intensity at 455 nm observed in a refence experiment under the same conditions but in the absence of POV6.








**Figure S13. Top**: Fluorescence spectra upon photoexcitation at 310 nm of POV6 (1 × 10^–4^ M) and disodium terephthalate (1 × 10^–4^ M) in water measured after different illumination times. **Bottom**: Corresponding fluorescence intensity *vs.* illumination time profiles at 425 nm (black dots). The red dots represent the fluorescence intensity 425 nm observed in a refence experiment under the same conditions but in the absence of POV6.

1. **XPS data**

POV6 was deposited on gold-metalized silicon wafers by spin-coating. To prepare the substrate, the gold-plated silicon wafers were rinsed with 95%-vol. EtOH and subsequently cleaned with ozone for 60s. 150 µL of 0.2 mg ml^–1^ POV6 solution were dropped on the sample, while the rotation speed was set to 700 rpm for 60s.

The surface composition was explored by X-ray photoelectron spectroscopy (XPS, Kratos Ultra DLD). The measurements were performed at room temperature with a background pressure of 1 × 10^–7^ Pa. For excitation, monochromic Al Kα radiation at 1486.6 eV was used. The X-ray source was operated at 150 W. Photoelectrons were released from the 300 µm × 700 µm analysis area and analyzed under normal incidence configuration. The photoelectron yield was enhanced applying a magnetic immersion lens. To avoid sample charging, a neutralizer supplied thermal electrons to the analysis area. A retarding lens system coupled by a hemispherical analyser with 40 eV pass energy was used for energy separation. The energy-filtered photoelectrons were amplified passing a microchannel plate and recorded by a delay line detector. The UNIFIT 2023 software was used for spectra analysis and composition calculation considering the specific transmission correction of the XPS machine. For curve fitting convolved Gaussian-Lorentzian peak profiles were simultaneously optimized with a Shirley background profile. The spectral calibration was performed by the adventitious carbon peak in the C 1s core-level spectrum at 285.0 eV. The core-levels of V 2p and O 1s were fitted together to account for their overlapping. Because of the Coster-Kronig effect the linewidth of V 2p_1/2_ was about 2x the linewidth of V 2p_3/2_. The peak area ratio V 2p_1/2_ / V 2p_3/2_ was kept at 1:2 and the V 2p_1/2_ - V 2p_3/2_ peak splitting at 7.33 eV. The V^(V)^ and V^(IV)^ oxidation state peaks are overlapping strongly. The best fit value for the V^(V)^ - V^(IV)^ peak separation is 0.95 eV. The binding energy of the V^(V)^ peak is situated at 517.3 eV. The peak width of the V^(V)^ peak is quite sharp with 1.1 – 1.3 eV, whereas the V^(IV)^ peak is broader with 1.6 – 1.8 eV due to the multiplet splitting resulting from the unpaired valence electron. Those peak fit parameters are in good agreement with NIST values.

The load-lock chamber of the XPS instrument was equipped with a UVC source (RBD instruments) providing light at 185 nm with an irradiance of 350 µW/cm². POV6 samples were UVC-treated for 10 minutes under vacuum (7 × 10^–5^ Pa). After illumination the samples were directly transferred into the XPS measurement chamber without breaking the vacuum.


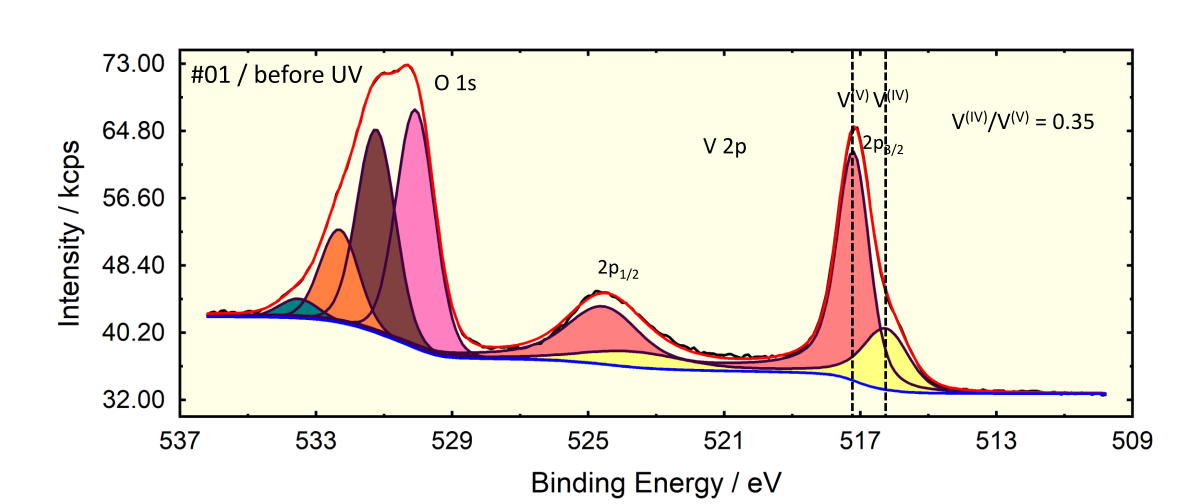


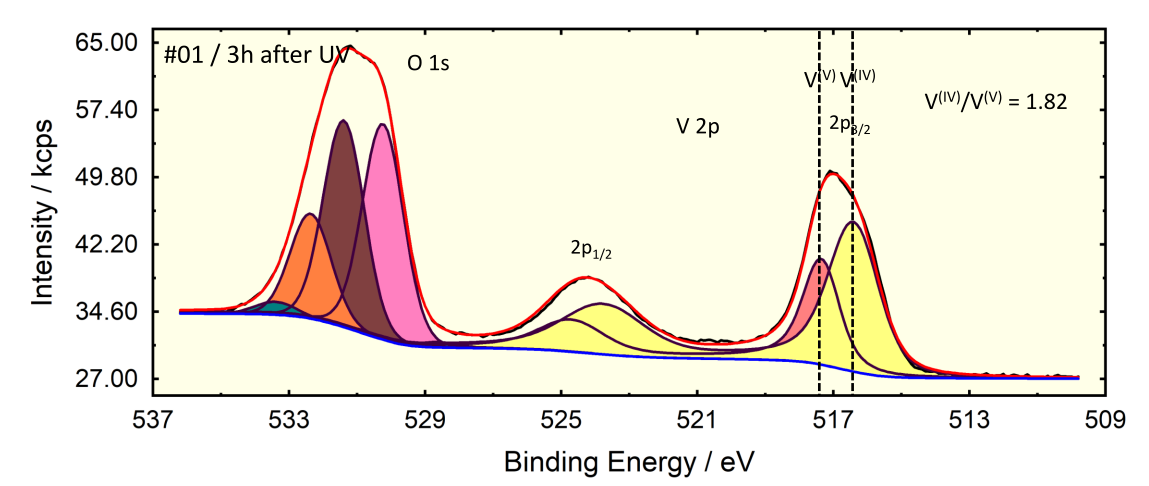


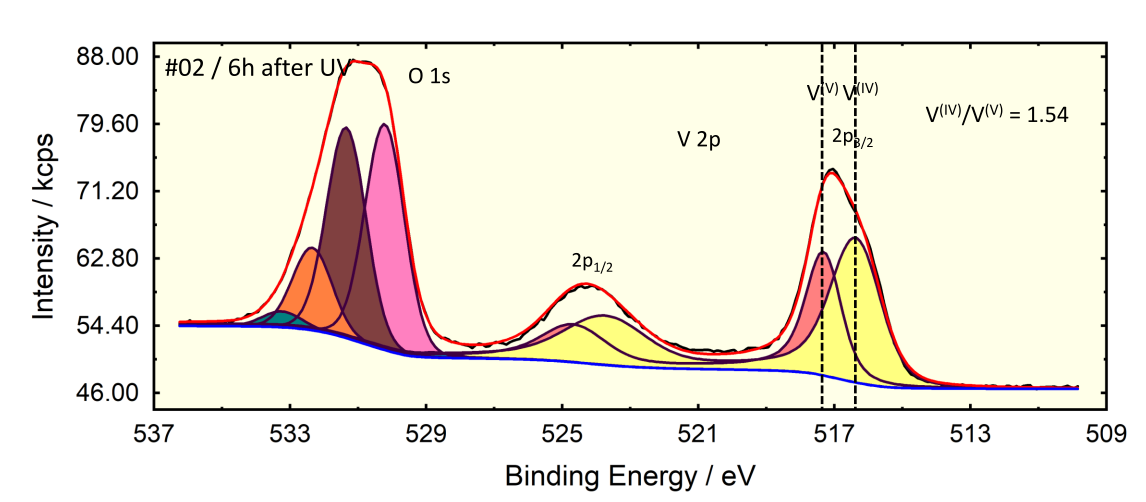


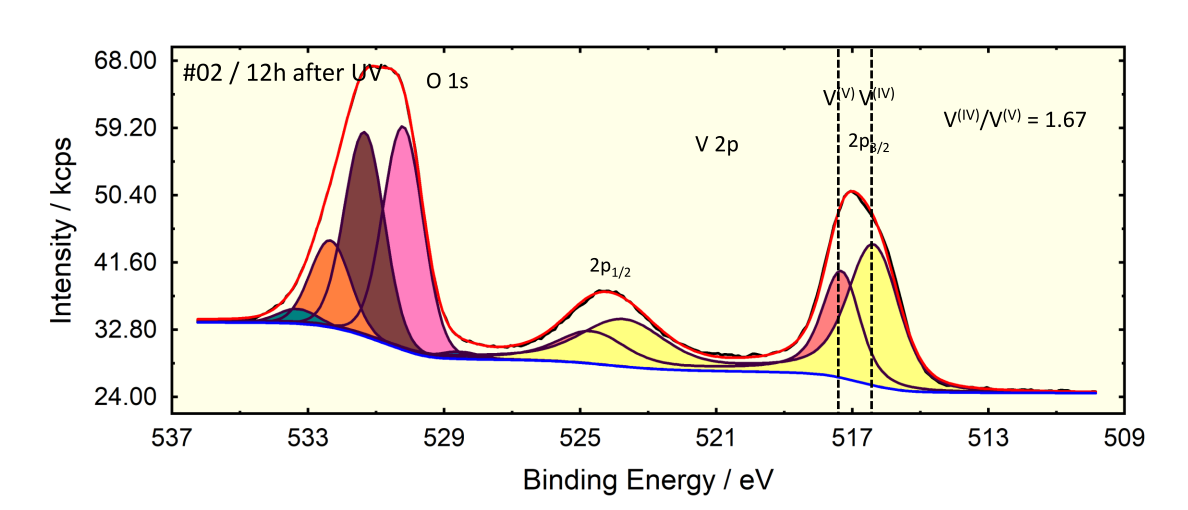


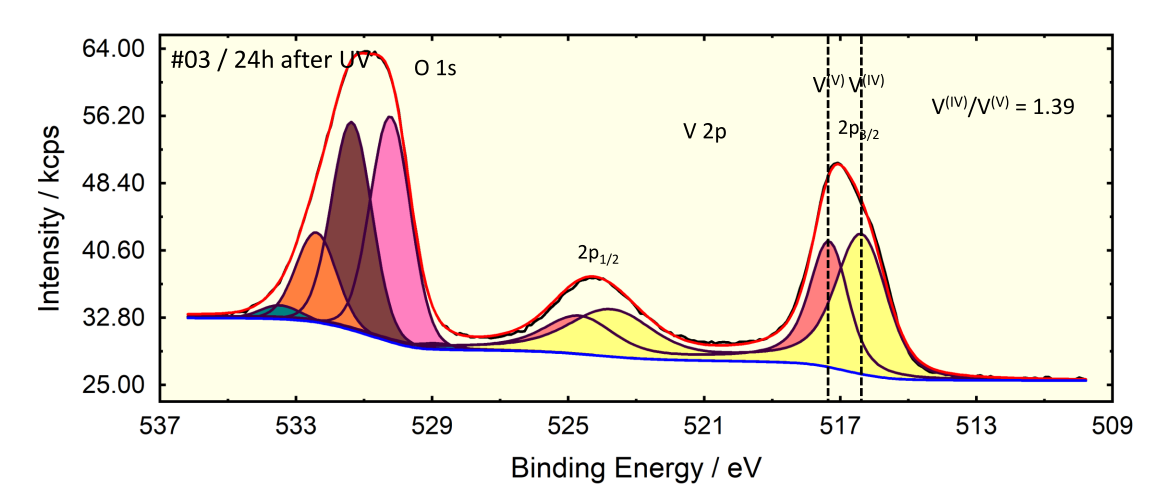


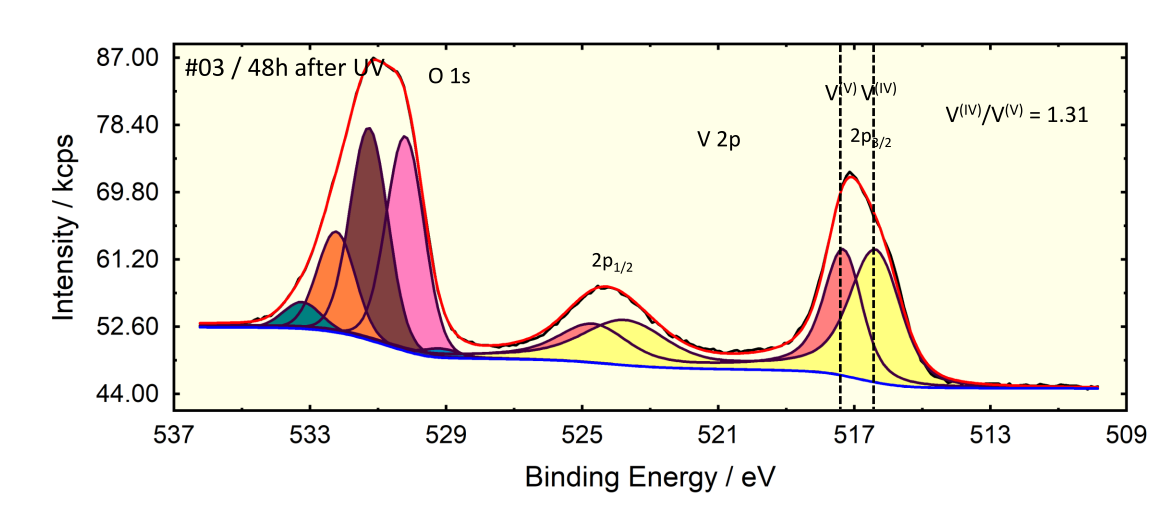


**Figure S14.** From Top to Bottom, XPS spectra of dried POV6 on gold substrate in a time period of 3 h, 6 h, 12 h, 24 h and 48 h after irradiation. The samples were irradiated with the 185 nm UV source for 10 min. Since the X-ray measurement itself also leads to a reduction of V^5+^ to V^4+^, every time period was measured on a separated sample to minimize the influence of the measurement process itself.

1. **References**

[1] S. Stoll, A. Schweiger, *J. Magn. Reson.* **2006**, *178*, 42–55.

[2] A. Schweitzer, T. Gutmann, M. Wächtler, H. Breitzke, A. Buchholz, W. Plass, G. Buntkowsky, *Solid State Nuclear Magn. Reson.* **2008**, *34*, 52–67.

[3] S. G. J. van Meerten, W. M. J. Franssen, A. P. M. Kentgens, *J. Magn. Reson.***2019**, *301*, 56–66.

[4] F. Neese, *WIREs Comput. Mol. Sci.* **2012**, *2*, 73–78.

[5] F. Weigend, R. Ahlrichs, *Phys. Chem. Chem. Phys.* **2005**, *7*, 3297–3305.

[6] M. Reiher, O. Salomon, B. Artur Hess, *Theor. Chem. Acc.* **2001**, *107*, 48–55.

[7] V. Barone, M. Cossi, *J. Phys. Chem. A* **1998**, *102*, 1995–2001.

[8] A. V. Marenich, C. J. Cramer, D. G. Truhlar, *J. Phys. Chem. B* **2009**, *113*, 6378–6396.

[9] T. Yanai, D. P. Tew, N. C. Handy, *Chem. Phys. Letters* **2004**, *393*, 51–57.

[10] F. Weigend, *Phys. Chem. Chem. Phys.* **2006**, *8*, 1057–1065.

[11] F. Neese, F. Wennmohs, A. Hansen, U. Becker, *Chem. Phys.* **2009**, *356*, 98–109.

[12] A. Kumar, J. A. Walker, D. M. Bartels, M. D. Sevilla, *J. Phys. Chem. A* **2015**, *119*, 9148–9159.
